# PD-1–targeted IL-15 mutein activates CD8^+^ and CD4^+^ T cells in infection and cancer

**DOI:** 10.1172/jci.insight.198701

**Published:** 2026-04-30

**Authors:** Isaraphorn Pratumchai, Marie Bernardo, Julien Tessier, Jaroslav Zak, Kristi L. Marquardt, Joon Sang Lee, Maheeka Bimal, AHyun Choi, Anthony M. Byers, Mikielia G. Devonish, Roberto Carrio, Dan Lu, Stella Martomo, Jeegar Patel, Yu-an Zhang, Ingeborg M. Langohr, Virna Cortez-Retamozo, Dinesh S. Bangari, Angela Hadjipanayis, Xiangming Li, Valeria R. Fantin, Donald R. Shaffer, John R. Teijaro

**Affiliations:** 1 The Scripps Research Institute, Department of Immunology and Microbiology, La Jolla, California, USA.; 2 Sanofi, Cambridge, Massachusetts, USA.; 3 Sanofi Vaccines, Orlando, Florida, USA.; 4 Kadmon Corporation, A Sanofi Company, New York, New York, USA.

**Keywords:** Immunology, Oncology, Cancer immunotherapy, Cytokines, Immunotherapy

## Abstract

Immune checkpoint inhibitors have transformed cancer therapy, yet many patients fail to achieve durable responses due to insufficient T cell reinvigoration. Cytokines offer promise for enhancing immunotherapy, but their clinical use is limited by toxicity and a narrow therapeutic index. Immunocytokines, engineered fusion proteins combining antibody specificity with cytokine activity, aim to overcome these challenges by targeting cytokine delivery to immune cells or the tumor microenvironment. We describe SAR445877 (SAR’877), a potentially novel PD-1–targeted immunocytokine that fuses a high-affinity anti–PD-1 antibody with a detuned IL-15/IL-15Rα sushi domain complex. SAR’877 blocks PD-1/PD-L1 and PD-1/PD-L2 interactions while selectively delivering IL-15 signals to PD-1^+^ T cells, enhancing proliferation and activation of antigen-experienced CD8^+^ and CD4^+^ T cells and NK cells, while minimizing systemic inflammation. Mechanistically, SAR’877 activates STAT5 signaling in PD-1^+^ lymphocytes and restores effector function in exhausted T cells. In preclinical models, a murine surrogate of SAR’877 accelerated viral clearance and induced robust antitumor immunity by expanding cytotoxic CD8^+^ T cells and promoting Th1 polarization. Notably, SAR’877 outperformed anti–PD-1 plus untargeted IL-15, highlighting the therapeutic potential of targeted IL-15 delivery. These findings position SAR’877 as a promising next-generation immunotherapy with enhanced efficacy and reduced cytokine-associated toxicities.

## Abstract 2

Next-generation anti-PD-1/IL-15 immunocytokine selectively delivers IL-15 signals to PD-1⁺ T cells, promoting proliferation and activation of antigen-experienced T cells, enhancing efficacy and reducing cytokine-associated toxicities

## Introduction

Cytokines, such as IFN-α and IL-2, were among the first modern immunotherapy drugs approved to treat cancer, marking a major milestone in immune-stimulating treatments for cancer (1). However, despite early excitement for these therapies, their use has been restrained due to dose-limiting toxic side effects associated with systemic administration and the advent of treatments with more favorable therapeutic profiles. Immune checkpoint blockade (ICB) has transformed treatment outcomes for patients with cancer by eliciting durable tumor regressions in the absence of many of the severe toxicities associated with high-dose IL-2 therapy (2). Still, ICB only works in a fraction of patients, and substantial unmet medical need persists for patients with cancers that are refractory to treatment (primary resistance) or who experience an initial response followed by relapse (acquired resistance). Translational studies have shown the relative fitness of T cells in patient tumors is a key predictor of response to ICB, with the presence of partially exhausted TCF1^+^ T cells predicting better clinical outcomes than tumors that show more terminally exhausted TCF1^–^PD-1^+^ T cells (3).

With the success and limitations of ICB realized, substantial efforts have concentrated on understanding how cytokine therapies could combine with ICB to maximize response or rescue resistant patients. Mechanistically, a study in mice chronically infected with lymphocytic choriomeningitis virus (LCMV) demonstrated that ICB combined with IL-2 acts upon TCF1^+^ T cells to generate a significantly greater number of transcriptionally distinct CD8^+^ effector T cells with better ability to clear viral infection than ICB alone (4). Nevertheless, the poor drug-like properties (i.e., short half-life, pleiotropic effects, and systemic toxicities) of IL-2 for cancer therapy have necessitated various engineering strategies aimed at overcoming these limitations for patients with cancer. Reduction of potency, extention of half-life, conditional activation, and antigen-directed targeting are all being explored (5). Like IL-2, IL-15 has demonstrated beneficial effects on lymphocyte and NK cell activation stimulating the proliferation and activation of CD8^+^ T cells and NK cells, leading to robust cytokine release and effector responses, which can promote control of viral infection and tumors. Despite these favorable properties, the short serum half-life of IL-15 necessitates frequent high-dose administrations to maintain therapeutic levels, thereby amplifying off-target effects and exacerbating systemic toxicity. IL-15 also triggers the production of downstream cytokines, including IFN-γ, IL-6, and TNF-α, further contributing to systemic inflammation. Consequently, systemic IL-15 administration has been associated with thrombocytopenia, hypotension, cytokine release syndrome and macrophage activation syndrome (6). These limitations underscore the need for alternative delivery formats for IL-15 therapy.

Here we describe SAR445877 (SAR’877), an antibody-cytokine fusion designed to block PD-1/PD-L1 and PD-1/PD-L2 interactions while delivering a mutated IL-15 selectively to PD-1^+^ T cells. SAR’877 exhibits strong binding to PD-1, effectively blocks PD-1 engagement, and preferentially stimulates proliferation and activation of antigen-experienced CD8^+^ T cells, CD4^+^ T cells, and NK cells with reduced inflammatory cytokine release compared with WT IL-15. The molecule’s activity depends on PD-1 expression and selectively enhances STAT5 signaling in PD-1^+^ lymphocytes. In vitro, SAR’877 reversed T cell exhaustion and boosted effector cytokine production. In mice, a murine surrogate of SAR’877 promoted faster viral clearance and rejuvenated exhausted T cells in the chronic LCMV clone-13 model. The murine surrogate of SAR’877 significantly enhanced antitumor immunity in B16-F10-OVA and CT26 tumor models by expanding effector CD8^+^ T cells, increasing Th1 polarization, and upregulating cytotoxic gene expression programs. Importantly, SAR’877 showed superior antitumor efficacy compared with anti–PD-1 plus untargeted IL-15 combinations, highlighting PD-1–targeted IL-15 delivery as a promising strategy to potentiate ICB while minimizing systemic cytokine toxicity.

## Results

### SAR’877 prevents T cell inhibition by antagonizing PD-1.

**Figure 1:**
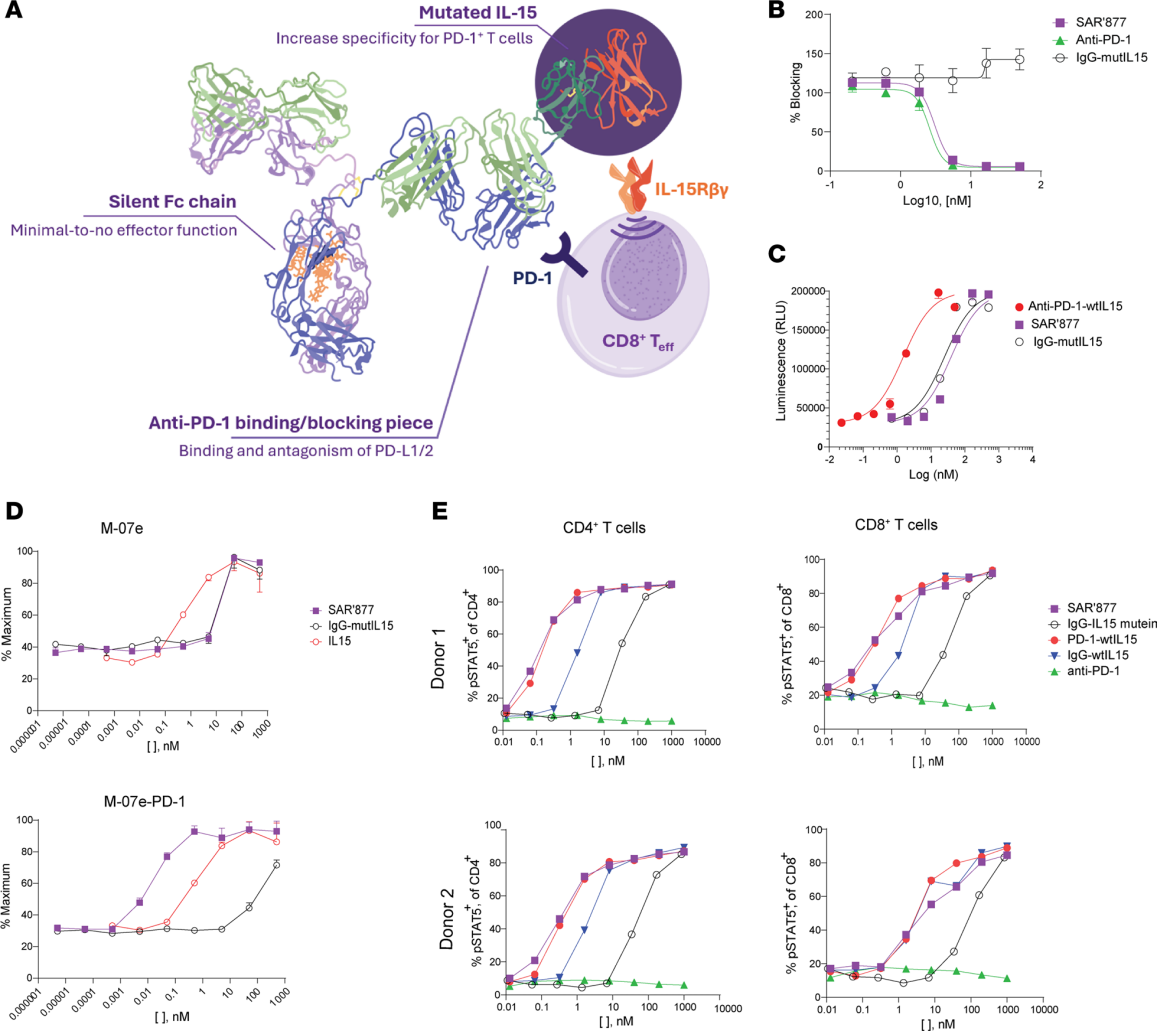
Functional activity of SAR’877. (**A**) SAR’877 design schematic. (**B**) PD-1/PD-L1 blockade measured by ELISA. (**C**) M07e cell proliferation following 3-day incubation with SAR’877 or control constructs (CTG assay). (**D**) Proliferation of parental (PD-1^–^) and PD-1^+^ M07e cells after 4-day incubation with immunocytokines (CTG assay; triplicate measurements). SAR’877 and IgG–mutIL-15 show equivalent potency in PD-1^–^ cells, whereas SAR’877 is markedly more potent in PD-1^+^ cells, confirming cis-targeted activity. (**E**) pSTAT5 induction in prestimulated human T cells treated with immunocytokines for 15 minutes (flow cytometry; 2 representative donors of 4 tested).

We generated SAR’877 by N-terminal fusion of a mutated variant of IL-15 bound to the sushi domain of IL-15Rα (referred to as mutIL-15) with a fully human anti–PD-1 antibody. SAR’877 is designed to facilitate delivery of IL-15 to PD-1^+^ T cells, resulting in superior proliferation and activation of tumor antigen-experienced CD8^+^ T cells capable of mediating antitumor activity (Figure 1A).

We characterized binding and functional activity of the antibody-cytokine fusion using in vitro assays. SAR’877 exhibited potent binding to human PD-1 protein (Supplemental Figure 1A; supplemental material available online with this article; https://doi.org/10.1172/jci.insight.198701DS1). The extent of SAR’877 binding was equivalent to the anti–PD-1 antibody without cytokine fusion (anti–PD-1), as well as anti–PD-1 bound to WT IL-15 (anti–PD-1/wtIL-15). However, mutIL-15 fused to a nonspecific IgG (IgG–IL-15) showed no association with anti–PD-1. SAR’877 efficiently blocked the binding of human PD-1 to human PD-L1 in a dose-dependent manner and with similar potency compared with anti–PD-1 (Figure 1B). To examine the consequences of PD-1 blockade, an in vitro functional assay in which artificial antigen-presenting cells (APCs) overexpressing PD-L1 are cocultured with T cells expressing an activation induced luciferase reporter was used. SAR’877, anti–PD-1/wtIL-15 and anti–PD-1 each exhibited dose-dependent induction of T cell activation, consistent with reduced suppression through PD-1 engagement (Supplemental Figure 1B). A similar functional effect was observed when blocking the interaction between PD-1 and PD-L2 (Supplemental Figure 1C).

### SAR’877 stimulates T cell proliferation with reduced potency compared with wtIL-15.

We next investigated the activity of the cytokine moiety of SAR’877. Human IL-15 binds to the IL-2Rβ chain, which recruits IL-2Rγ chain to form a heterodimeric receptor and initiate signaling through the JAK/STAT pathway (7). IL-2Rβγ activation and downstream signaling results in lymphocyte proliferation and promotes cytotoxic effector function. SAR’877 IL-15 mutein contains a single amino acid mutation intended to decrease the potency of stimulation through IL-2Rβγ receptor. SAR’877 exhibited weak binding to HEK293 cells engineered to express human IL-2Rβ but lacking expression of PD-1 (Supplemental Figure 1D). Binding of SAR’877 was similar to IgG–mutIL-15, both of which were strongly reduced compared with anti–PD-1/wtIL-15. SAR’877 and IgG–mutIL-15 stimulated proliferation of human M07e leukemia cells (Figure 1C) as well as primary human peripheral blood mononuclear cells (PBMCs), including CD4^+^ and CD8^+^ T cells, in a dose-dependent manner (Supplemental Figure 1, E and F). Importantly, SAR’877 was less potent when compared with anti–PD-1/wtIL-15, demonstrating weaker activity of the mutated cytokine moiety.

The Fc region of SAR’877 is mutated to reduce activity through complement-dependent cytotoxicity (CDC) and antibody-dependent cytotoxicity (ADCC). SAR’877 and anti–PD-1 exhibit minimal binding to complement protein and minimal induction of CDC and ADCC compared with antibodies containing unmodified Fc regions (Supplemental Figure 1G). Together these results demonstrate that SAR’877 blocks PD-1–mediated immunosuppression, promotes lymphocyte proliferation through its mutIL-15 moiety, and exhibits minimal activity through CDC and ADCC.

### SAR’877 activity is enhanced by expression of PD-1.

To explore the effect of PD-1 expression on the activity of SAR’877, we engineered M07e cells to express human PD-1 protein and compared the activity of antibody-cytokine fusions in parental cells and PD-1^+^ engineered cells. M07e cells lacking PD-1 expression proliferated weakly in response to treatment with SAR’877 (Figure 1D). In the absence of PD-1 expression, there was no difference in activity of SAR’877 and IgG–mutIL-15, and both constructs were less potent compared with human WT, recombinant IL-15. In contrast, on PD-1^+^ M07e cells, the potency of SAR’877 was markedly higher (EC_50_ = 0.015 nM) compared with the nontargeted control IgG–mutIL-15 for which the EC_50_ was not quantifiable, confirming that SAR’877 activity is partially mediated by PD-1 expression (Figure 1D, panel 2). The effect of PD-1 expression on SAR’877 activity was further explored in primary human immune cells. Human T cells were isolated from PBMCs, prestimulated with a CD3/CD28 agonist, and treated with SAR’877 or various control antibody-cytokine fusions. Stimulation through IL-2Rβγ was measured using flow cytometry to detect phosphorylated STAT5 (pSTAT5). T cells that were preactivated and rested exhibit upregulation of surface PD-1 expression (Supplemental Figure 1H). Anti–PD-1 antibody on its own had no effect on pSTAT5, while SAR’877 induced pSTAT5 on both CD4^+^ and CD8^+^ T cells (Figure 1E). Strikingly, SAR’877 was 203-fold more potent compared with the nontargeted cytokine IgG–mutIL-15. SAR’877 activity overlapped with that of anti–PD-1/wtIL-15, demonstrating that expression of PD-1 compensates for the weaker activity of mutIL-15.

In a separate assay, we mixed M07e cells together with PD-1^+^ M07e cells at a 1:1 ratio and then added SAR’877, control IgG–mutIL-15, or recombinant IL-15 and measured pSTAT5. While antibiotic and clonal selection caused a higher overall pSTAT5 signal in our PD-1^+^ M07e cells, we observed that SAR’877 induced significantly higher levels of pSTAT5 than the control IgG–mutIL-15 (Supplemental Figure 1I). Indeed, SAR’877 induced nearly as much pSTAT5 as recombinant IL-15, whereas IgG–mutIL-15 showed only background levels of pSTAT5 at all but the 2 highest concentrations. In contrast, SAR’877 and control IgG–mutIL-15 showed overlapping pSTAT5 activation on PD-1^–^ M07e cells. Thus, these results demonstrate that SAR’877 delivers IL-15–mediated stimulation through *cis*-targeting of PD-1^+^ cells and that PD-1 expression is critical to its functional activity.

The functional activity of SAR’877 was explored further in human primary immune cells. Human PBMCs were treated with SAR’877 for 6 days, and proliferation was measured through detection of Ki-67^+^ by flow cytometry. SAR’877 promoted proliferation of CD4^+^ T cells, CD8^+^ T cells, and NK cells (Supplemental Figure 1J). The strongest activity was observed in NK cells, which reached nearly 100% of cells proliferating, followed by CD8^+^ T cells, while effects in CD4^+^ T cells were limited. Since induction of proinflammatory cytokines may contribute to toxicity of cytokine therapies, the effect of SAR’877 on cytokine secretion was explored. PBMCs treated with SAR’877 secreted IFN-γ with virtually no secretion of IL-6 and TNF-α, in contrast to human recombinant IL-15, which induced high levels of all 3 cytokines (Supplemental Figure 1K). The effects of SAR’877 on activation of antigen-specific T cell responses were explored. PBMCs were treated with CD4 and CD8 antigen peptide pools in the presence of drug, and activation was measured through secretion of IFN-γ. SAR’877 stimulated IFN-γ release from both CD4^+^ and CD8^+^ T cells, with stronger activity compared with the control IgG–mutIL-15 and anti–PD-1 alone (Supplemental Figure 1L). To investigate function on NK cells, human NK cells were cocultured with K562 target cells in the presence of SAR’877 for 5 days, and target cell viability was monitored. SAR’877 and IgG–mutIL-15 similarly stimulated NK cell cytotoxicity against K562 target cells, while anti–PD-1 had no effect (Supplemental Figure 1M). Thus, SAR’877 stimulates proliferation, activation, and cytotoxic activity of CD4^+^ and CD8^+^ T cells as well as NK cells.

### SAR’877 reverses T cell exhaustion in vitro.

**Figure 2:**
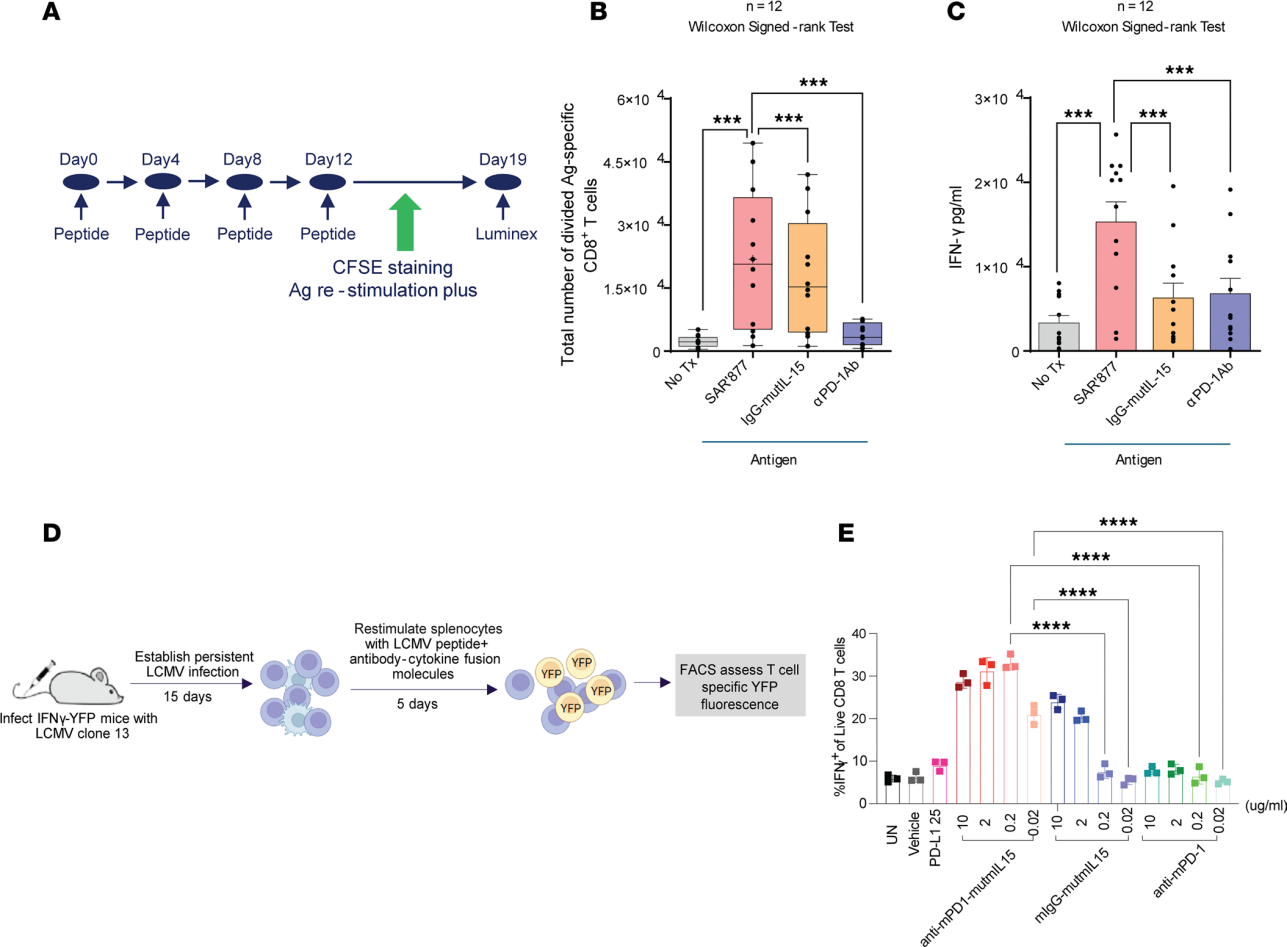
SAR’877 and anti–mPD-1–mutmIL-15 reverse CD8^+^ T cell exhaustion *in vitro*. (**A**) Schematic of the MIMIC human CD8^+^ T cell exhaustion assay. (**B** and **C**) Antigen-specific CD8^+^ T cell numbers and IFN-γ production following treatment with SAR’877 (10 mg/mL) or controls (*n* = 12 donors). Paired comparisons between treatment conditions were analyzed using the Wilcoxon signed-rank test. Data are shown as box plots with median and interquartile range. ***P < 0.001. (**D**) Schematic of the murine ex vivo T cell restimulation assay. (**E**) IFN-γ^+^ cell frequency among exhausted CD8^+^ T cells isolated from IFN-γ–YFP mice with chronic LCMV infection, following culture with anti–mPD-1–mutmIL-15 or controls (flow cytometry). Statistical significance was determined using one-way ANOVA followed by Šídák’s multiple comparisons test. Data are presented as bar plots showing mean ± SD. ****P < 0.0001.

Our experiments suggest that SAR’877 preferentially stimulates PD-1–expressing immune cells. To understand how SAR’877 may modulate lymphocytes in the context of cancer, we investigated the expression of its target proteins using scRNA-seq data. Previously published datasets of tumor-infiltrating cells isolated from human lung, ovarian, colorectal, breast, and esophageal tumors were pooled and analyzed using the BioTuring Talk2Data (8). *IL2RB* and *IL2RG* genes were expressed in CD4^+^ T cells, CD8^+^ T cells, and NK cells, consistent with the known functions of the IL-2R (Supplemental Figure 2A). *PDCD1* was limited to certain T cell subsets, including effector T cells and exhausted T cells. Having observed prominent expression of both PD-1 and IL-2 receptor in exhausted T cells, we explored the potential activity of SAR’877 on exhausted T cells in vitro. We utilized the human Modular Immune In vitro Construct (MIMIC) CD8^+^ T cell exhaustion assay previously described in the literature (9), in which in vitro derived dendritic cells (iDCs) are cocultured with autologous CD8^+^ T cells and repeatedly stimulated with antigen (Figure 2A). After repeated antigen stimulation, CD8^+^ T cells exhibit an exhausted phenotype characterized by diminished capacity to proliferate and reduced secretion of IFN-γ (Supplemental Figure 2B). Exhausted T cells also show elevated expression of PD-1 compared with unstimulated cells and functional T cells stimulated with antigen, consistent with the expression data from human tumor-infiltrating lymphocytes (TILs) (Supplemental Figure 2C). Exhausted CD8^+^ T cells were treated with SAR’877 or controls, followed by assessment of their proliferation and activation status. Exhausted CD8^+^ T cells treated with SAR’877 exhibited significantly increased proliferation upon antigen restimulation compared with untreated cells (Figure 2B). While both IgG–mutIL-15 and anti–PD-1 were also capable of stimulating proliferation, the effect was significantly lower compared with SAR’877. Similarly, exhausted CD8^+^ T cells treated with SAR’877 produced significantly higher levels of IFN-γ when compared with cells treated with either IgG–mutIL-15 or anti–PD-1 (Figure 2C). SAR’877 restores key functions of exhausted CD8^+^ T cells by promoting their proliferative capacity and enabling distinct expression of functional cytokines such as IFN-γ, 2 critical features typically lost during T cell exhaustion. Exhaustion marker surface staining performed at the end of the assay (day 19) revealed some minor decreases in PD-1, TIM-3, and LAG-3 median fluorescence intensity (MFI) after SAR’877 treatment relative to controls or other treatments (data not shown).

We next sought to explore the activity of SAR’877 on exhausted T cells by leveraging a well-established model of chronic viral infection. C57BL/6J mice exposed to LCMV clone-13 develop persistent viral infection and develop dysfunctional, exhausted CD4^+^ and CD8^+^ T cells (10). Since SAR’877 does not cross react with mouse PD-1, we generated a murine surrogate version by fusing mutated mouse IL-15 (mutmIL-15) to a mouse anti–PD-1 antibody (anti–mPD-1). The functional activity of the surrogate molecule (herein referred to as anti–mPD-1–mutmIL-15 or PD-1–targeted IL-15 mutein) was characterized in in vitro assays. Anti–mPD-1–mutmIL-15 blocked interaction of murine PD-1 with its ligand PD-L1 (Supplemental Figure 2D) and stimulated proliferation of murine T lymphocyte cell line CTLL-2 (Supplemental Figure 2E). Like the human construct, anti–mPD-1–mutmIL-15 was less potent at stimulating lymphocyte proliferation compared with anti–PD-1 antibody fused to WT murine IL-15 (anti–PD-1–wtmIL-15) and, thus, demonstrated comparable activity to the human molecule. The ability of anti–mPD-1–mutmIL-15 to improve functions of exhausted CD8^+^ T cells was further tested using the in vitro T cell exhaustion assay as depicted in Figure 2D (11). Flow cytometry analysis following 5 days of in vitro stimulation of splenocytes from chronically LCMV-infected mice revealed that anti–mPD-1–mutmIL-15 not only strongly promoted IFN-γ production by CD8^+^ T cells, but it also enhanced expansion of TIM-3^–^ D^b^GP33^+^ antigen-specific CD8^+^ T cells while concurrently reducing PD-1 expression (Figure 2E and Supplemental Figure 2, F–K), demonstrating superior functional restoration compared with anti–mPD-1 or nontargeted mIgG–mutmIL-15.

### SAR’877 reverses T cell exhaustion in vivo.

**Figure 3:**
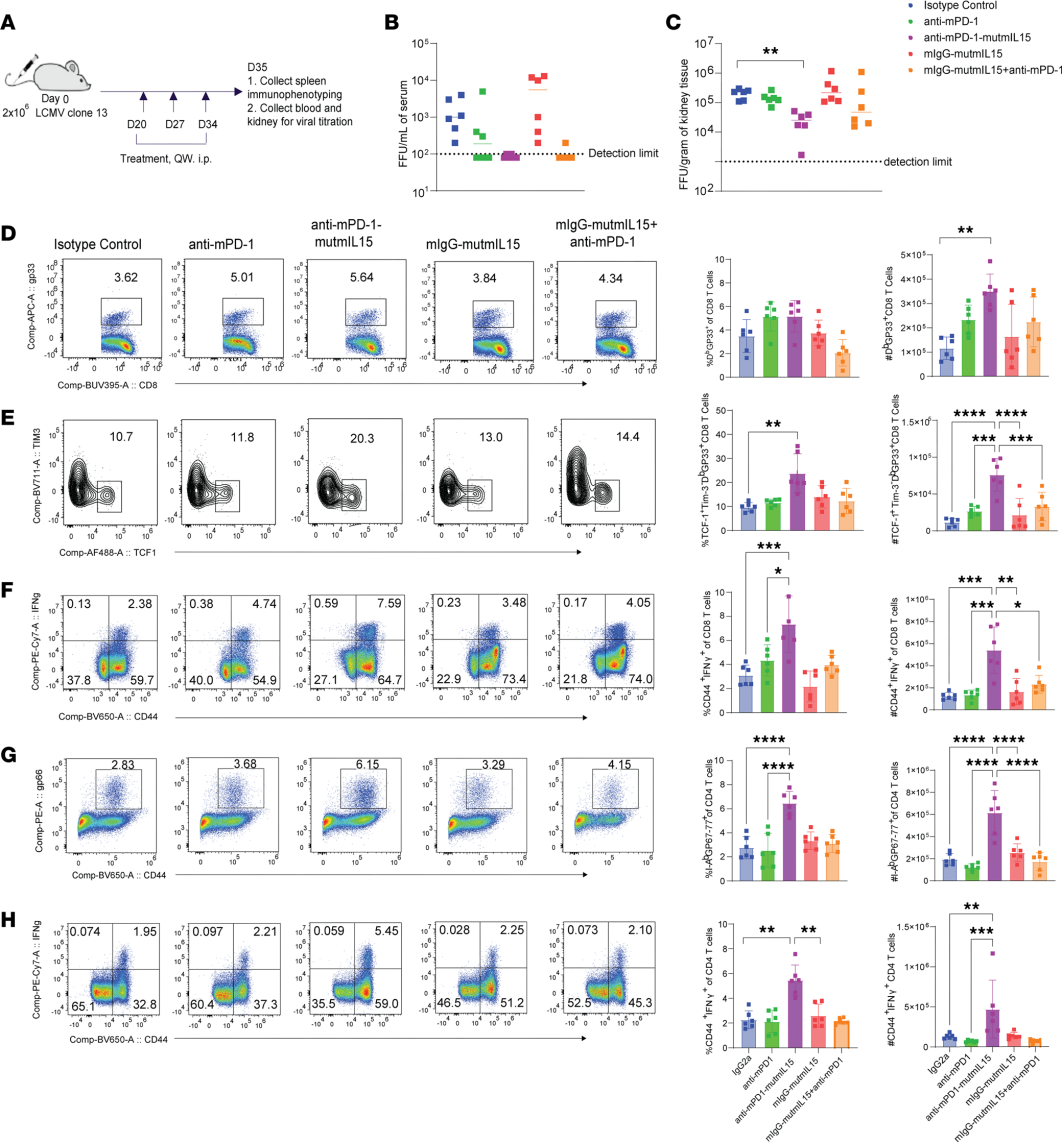
SAR’877 murine surrogate reverses T cell exhaustion in vivo and promotes clearance of persistent LCMV infection. (**A**) Schematic diagram of in vivo chronic LCMV infection model. (**B** and **C**) LCMV viral titer measured in serum and kidney of mice treated with 1 mg/kg anti–mPD-1–mutmIL-15 or controls at day 35 after infection. Mice harboring chronic LCMV infection were treated with antibody-cytokine fusions, and effects on immune cells in spleens were analyzed by flow cytometry. Anti–mPD-1–mutmIL-15 strongly increased expansion and activation viral specific T cells compared with anti–PD-1 or the nontargeted control cytokine mIgG–mutmIL-15. Data are shown as scatter dot plots with mean. Statistical significance was assessed using the Mann-Whitney *U* test. **P < 0.01. (**D**–**H**) Expansion of antigen-specific CD8^+^ T cells (**D**), expansion of antigen-specific TCF1^+^stem-like CD8^+^ T cells (**E**), expansion of IFN-γ-producing CD8^+^ T cells after *ex vivo* stimulation with LCMV-specific GP_33–41_ peptide (**F**), expansion of virus-specific CD4^+^ T cells (**G**), and (**P < 0.01) IFN-γ–producing CD4^+^ T cells (**H**) following ex vivo stimulation with LCMV-specific GP_61–80_ peptide stimulation were measured using flow cytometry. Experiments were carried out with *n* = 4–5 and repeated 3 times. Statistical significance was determined using 1-way ANOVA followed by Šídák’s multiple comparisons test. *P < 0.05, **P < 0.01, ***P < 0.001, ****P < 0.0001.

Given that CD8^+^ T cells play crucial roles in promoting antiviral immune response during chronic LCMV infection, we tested the in vivo function of the PD-1–targeted IL-15 mutein in promoting viral clearance in C57BL/6J WT mice persistently infected with LCMV CL13 (Figure 3A). Treatments with anti–mPD-1–mutmIL-15 promoted faster clearance of the virus from both serum and kidney compared with treatments with anti–mPD-1 or mIgG–mutmIL-15 (Figure 3, B and C). A combination of nontargeted mIgG–mutmIL-15 with anti–mPD-1 was able to effectively reduce viral titers in the serum but not the kidney (Figure 3C). Flow cytometry analyses of spleens at day 35 after infection revealed that in vivo treatments with anti–mPD-1–mutmIL-15 significantly increased antigen-specific CD8^+^ T cells and T precursor exhausted (Tpex) TCF1^+^TIM-3^–^CD8^+^ T cells compared with controls (Figure 3, D and E). In addition, ex vivo stimulation with LCMV-specific GP33 peptide showed that mice treated with the PD-1–targeted IL-15 mutein expressed markers indicative of effector function, including the degranulation marker CD107a, IFN-γ, TFN-α, and IL-2 compared with control treatments (Figure 3F and Supplemental Figure 3, A and B). Moreover, PD-1–targeted IL-15 mutein significantly increased antigen-specific CD4^+^ T cells and markedly improved their ability to produce IFN-γ upon ex vivo stimulation (Figure 3, G and H). Because both Tfh and Tregs express PD-1, we assessed whether our anti–mPD-1–mutmIL-15 molecule altered these cellular populations. We observed no significant change in either Tfh or Tregs compared with the isotype control with any of the treatments except the mIgG–mutmIL-15, which significantly increased Treg numbers (Supplemental Figure 3, C and D). Because IL-15 exerts significant effects on NK cell activation/differentiation, we also measured NK cell numbers in Clone-13–infected mice with all treatments. As anticipated, treatment with mIgG–mutmIL-15 or mIgG–mutmIL-15 ^+^ anti–PD-1 significantly increased NK cell numbers compared with the isotype control, and anti–mPD-1–mutmIL-15 modestly decreased NK cell numbers in spleen (Supplemental Figure 3E). We finally tested whether the CD8^+^ T cell compartment was solely responsible for the effects of anti–mPD-1–mutmIL-15 on reversing T cell exhaustion and promoting faster LCMV clearance by employing the in vivo CD4^+^ T cell depletion method as previously described (12). Notably, an intact-CD4^+^ T cell compartment was required for PD-1–targeted IL-15 mutein to expand GP33-specific CD8^+^ T cells (Supplemental Figure 3F). However, we did observe significant increases in IFN-γ^+^ GP33-41 specific CD8^+^ T cells (Supplemental Figure 3G). Furthermore, CD4^+^ T cell depletion also abolished the enhanced viral clearance in serum and lung tissues following anti–PD-1–mutmIL-15 (Supplemental Figure 3H). Thus, our data suggest that PD-1–targeted IL-15 mutein requires CD4^+^ T cell targeting for its functional outcomes.

**Figure 4:**
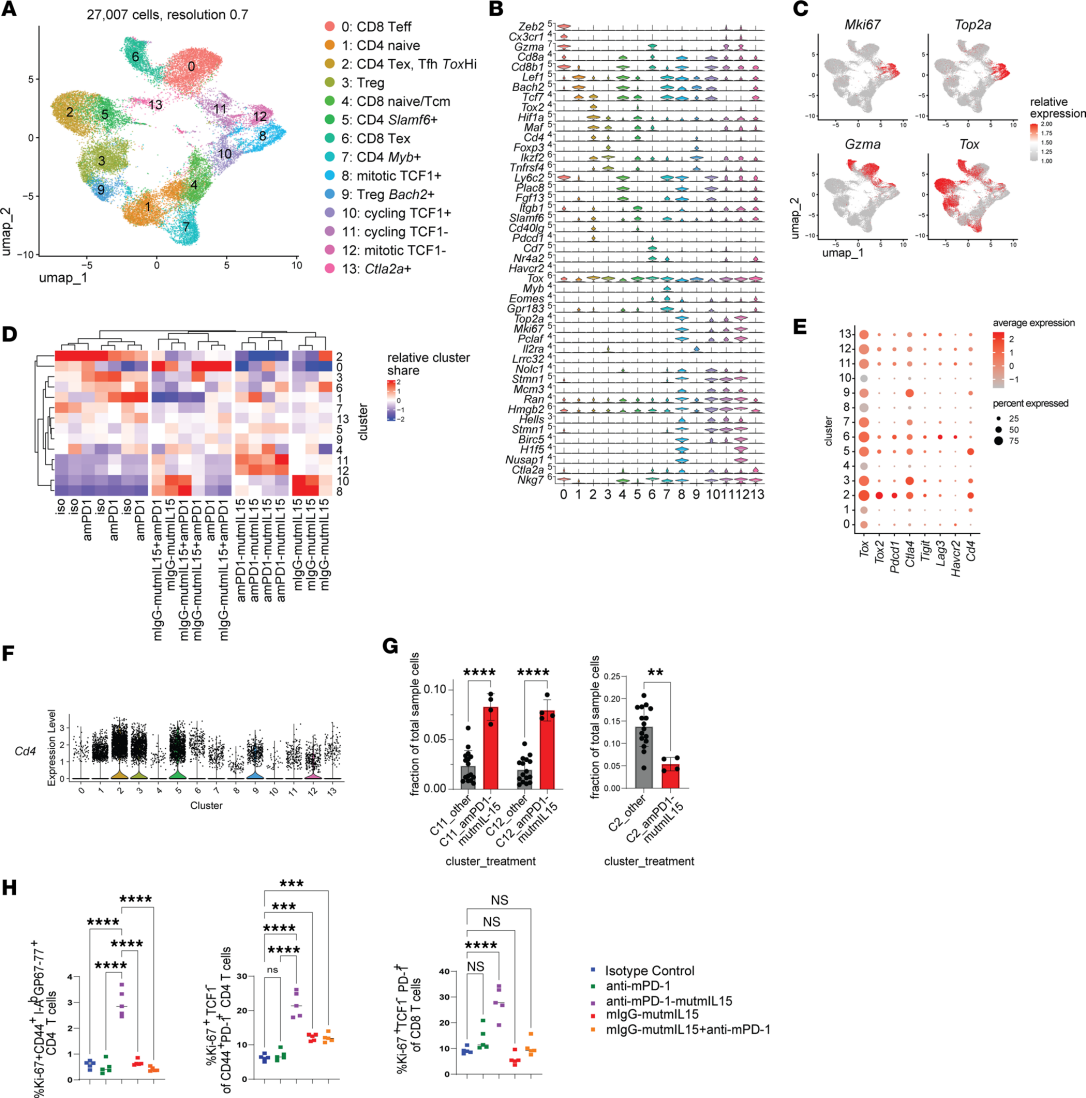
Single-cell transcriptomic analysis of anti–mPD-1–mutmIL-15 effects on T cells. C57BL/6 mice with chronic LCMV infection were treated with isotype control, anti–mPD-1, anti–mPD-1–mutmIL-15, mIgG–mutmIL-15, or mIgG–mutmIL-15 + anti–mPD-1. (**A**) UMAP clustering of pooled CD4^+^ and CD8^+^ T cells. (**B**) Violin plot of cluster marker genes. (**C**) Gene expression in UMAP space. (**D**) Relative cluster abundance per sample. (**E**) Dot plot of gene expression and cell fraction in Cluster 1. (**F**) CD4 expression across clusters. (**G**) Cell fractions in clusters C1, C11, C12 for anti–mPD-1–mutmIL-15 vs. other treatments Statistical significance was determined using a 2-tailed unpaired t test (**P = 0.0018). (**H**) Flow cytometry of Ki-67^+^ virus-specific CD4^+^ T cells and Ki-67^+^CD44^+^PD-1^+^TCF1^–^ CD4^+^/CD8^+^ T cells at day 30 after infection. Statistical significance was determined using 1-way ANOVA followed by Šídák’s multiple comparisons test. **P < 0.01, ***P < 0.001, ****P < 0.0001.

To better understand how the PD-1–targeted IL-15 mutein improves antiviral T cell responses, we analyzed splenic T cells in the LCMV model by scRNA-seq. Mice infected with LCMV clone-13 (Cl-13) were treated with anti–mPD-1–mutmIL-15, mIgG–mutmIL-15, mIgG–mutmIL-15 + anti–mPD-1, anti–mPD-1, or isotype control. Total splenic T cells were analyzed at day 30 after infection by scRNA-seq. Following removal of low-quality cells and doublets, clustering at resolution 0.7 produced 14 clusters including subset-specific clusters such as Tregs (C3) or naive/central memory CD8^+^ T cells (C4) but also mixed clusters defined by cell state such as mitotic CD4^+^ and CD8^+^ TCF1^lo^ T cells (C12) (Figure 4, A and B, and Supplemental Figure 4A). These cell identities were corroborated using whole-transcriptome–based cell identification by the SingleR package with the ImmGen ultra-low input RNA-seq dataset (GSE109125) as reference (Supplemental Figure 4A). There were 4 clusters of cycling T cells, 2 containing TCF1^+^ T cells (C8, C10) and 2 containing more highly differentiated TCF1^lo^ T cells (C11, C12). C11 primarily contains dividing effector CD8^+^ T cells characterized by *Gzma* expression (Figure 4, B and C).

Analyzing the relative fraction of T cells in each cluster by treatment revealed that PD-1–targeted IL-15 mutein uniquely enhanced the relative proportion of T cells in C11 and C12, whereas mIgG–mutmIL-15 enhanced C8 and C10 with both antibodies, reducing the relative fraction of C0 cells (Figure 4D). Interestingly, all the antibody-enhanced clusters contained actively cycling T cells, suggesting that anti–mPD-1–mutmIL-15 and mIgG–mutmIL-15 promote cycling of T cells. Specifically, the anti–mPD-1–mutmIL-15 enhanced C11 and C12 containing mostly S-phase TCF1^lo^ T cells (C11) and mitotic TCF1^lo^ T cells (C12), whereas mIgG–mutmIL-15 enhanced the mitotic TCF1^+^ T cells (C8) and G1 phase TCF1^+^ T cells (C10). The CD4^+^ T cell cluster C2, reduced in relative abundance by both antibodies, expressed the highest levels of inhibitory receptors and transcription factor *Tox* (Figure 4E). Of the dividing clusters, C11 and C12 also contained the highest percentage of CD4-expressing T cells, and the ratio of CD4/CD8a in C12 was increased in anti–PD-1–mutmIL-15–treated mice, suggesting the PD-1–targeted IL-15 mutein enhances the cycling of CD4^+^ T cells (Supplemental Figure 4B). Indeed, compared with other treatments, the fraction of C2 was significantly reduced in anti–mPD-1–mutmIL-15–treated mice, and the fraction of C11 and C12 significantly enhanced (Figure 4, F and G).

Examining the vectors estimated from RNA velocity data aligned with the identification of C2 as a cluster of terminally differentiated CD4^+^ T cells and C6 terminally differentiated, exhausted CD8^+^ T cells (Supplemental Figure 4C). The pattern of velocity vectors also supported the model of M-phase cluster C12 giving rise to TCF1^–^ T cells, whereas M-phase cluster C10 containing TCF1^+^ T cells mostly connected to other TCF1^+^ clusters (Supplemental Figure 4C). These observations suggest that anti–mPD-1–mutmIL-15 selectively enhanced the cycling of TCF1^lo^ T cells, whereas mIgG–mutmIL-15 selectively enhanced the cycling of TCF1^+^ T cells. The expression pattern of *Pdcd1* and pseudotime predicted from RNA velocity data supported the notion that cluster C2 contains terminally differentiated/exhausted CD4^+^ T cells expressing the highest levels of *Pdcd1*, and PD-1–targeted IL-15 mutein and nontargeted IL-15 mutein, thus, prevent the formation of these cells by promoting the cycling of less terminally exhausted CD4^+^ T cells (Supplemental Figure 4, D and E). Partition-based graph abstraction analysis and random walk analysis provided data consistent with the hypothesis that differentiated clusters C0 and C6, and CD4^+^ T cells clusters C5 and C2, originate primarily from the pool of cycling T cells in C12–C11, suggesting that these cells are undergoing antigen-induced expansion rather than homeostatic proliferation (Supplemental Figure 4, E and F). Consistently, flow cytometry analyses of total splenocytes from day 30 after infection showed that anti–mPD-1–mutmIL-15 strongly induced proliferation of virus-specific CD4^+^ T cells, TCF1^–^PD-1^+^CD4^+^ T cells, and TCF1^–^PD-1^+^CD8^+^ T cells compared with other treatment groups (Figure 4H). Complementary ex vivo stimulation of transferred P14 T cells revealed that TCF1^–^CD8^+^ T cells produced slightly more IFN-γ than TCF1^+^ cells, indicating that these proliferating TCF1^–^ cells retain effector function (Supplemental Figure 4D).

### PD-1 targeted IL-15 mutein exhibits potent antitumor activity in mouse tumor models.

**Figure 5:**
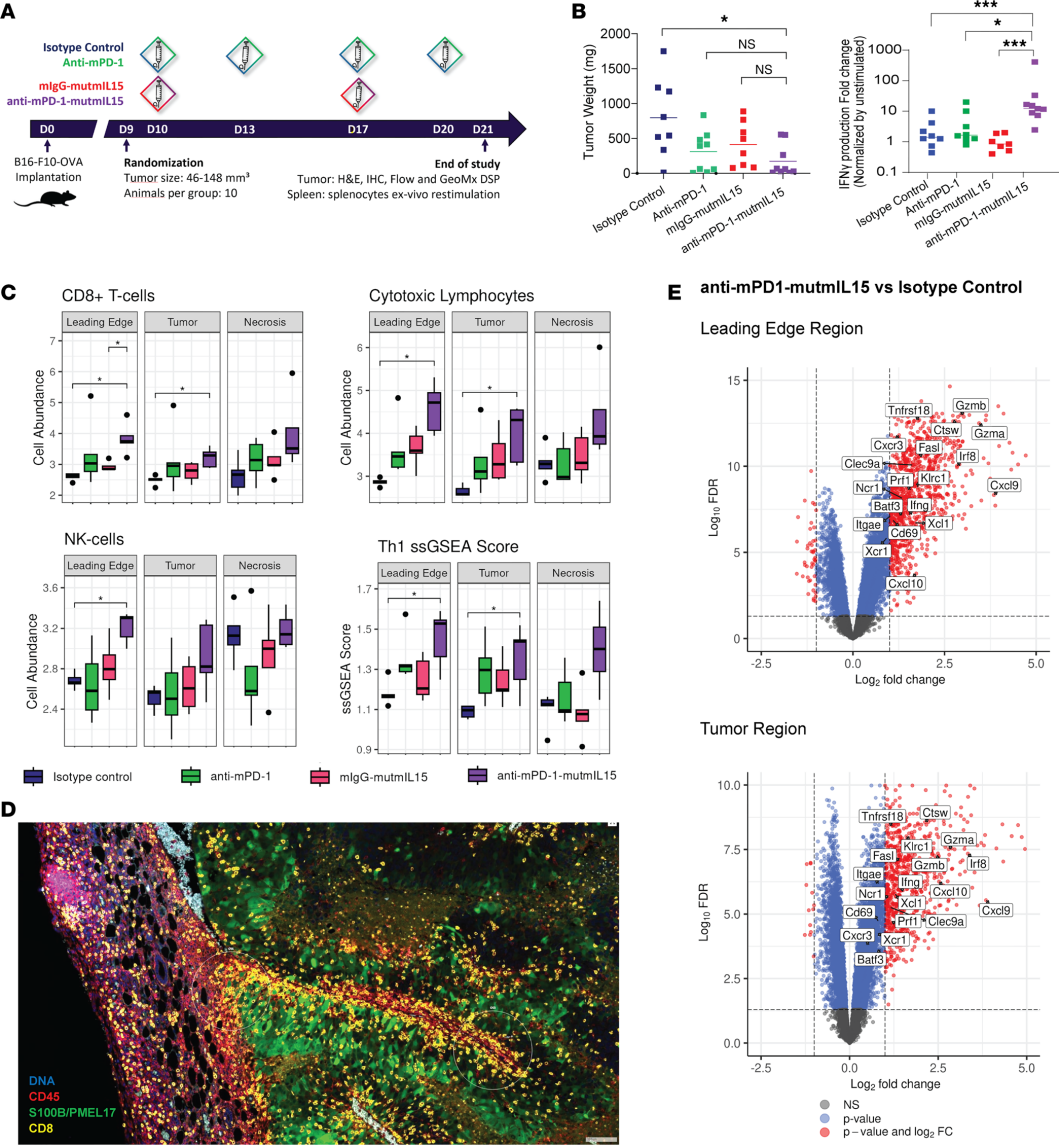
Anti–mPD-1–mutmIL-15 activity in murine B16-F10-OVA melanoma model. (**A**) Schematic of the B16-F10-OVA biomarker study. (**B**) B16-F10-OVA tumor weights at sacrifice and IFN-γ production by OVA-restimulated splenocytes 11 days after treatment. Data are shown as scatter dot plots with mean. Statistical significance was assessed by 1-way ANOVA followed by Dunnett’s multiple comparisons test versus anti–mPD-1–mutmIL-15. *P < 0.05; ***P < 0.001. (**C**) GeoMx DSP cell-type abundance (MCP-counter) and Th1 pathway enrichment (GSVA) by spatial compartment (Leading Edge, Tumor, Necrosis). Data are shown as box plots with median and interquartile range. *P < 0.05. (**D**) Representative GeoMx DSP image of CD8^+^ T cell infiltration in anti–mPD-1–mutmIL-15-treated tumors. Scale bar 100 μm. (**E**) GeoMx DSP volcano plots of DEGs in anti–mPD-1–mutmIL-15 vs. isotype control, per compartment. Group comparisons were performed using Wilcoxon rank-sum tests with FDR correction. FDR < 0.05 was considered statistically significant.

We next explored the activity of the PD-1–targeted IL-15 mutein in different syngeneic mouse tumor models. Antitumor efficacy was observed broadly across 12 murine tumor models, with 5 models showing complete responses in several mice and all 12 models achieving statistically significant antitumor efficacy compared with vehicle control (*P* < 0.05) (Table 1 and Supplemental Figure 5A). Treatment was generally well tolerated, with minimal to no weight loss observed (Supplemental Figure 5B). In the MC38 tumor model, PD-1–targeted IL-15 delivery did not compromise NK cell activity; the mouse surrogate of SAR’877 induced robust NK cell expansion, comparable with untargeted mutmIL-15, and significantly increased the frequency of functionally active NK cells, as evidenced by elevated granzyme B expression (Supplemental Figure 5C). These data demonstrate that, despite its design to preferentially engage PD-1^+^ T cells, SAR’877 preserves NK cell expansion and cytotoxic function in vivo, supporting both adaptive and innate cytotoxic immune responses. In contrast, PD-1–WT IL-15 induced rapid lethal toxicity in MC38 tumor-bearing mice, highlighting the narrow therapeutic window of unmodified IL-15. SAR’877, incorporating an attenuated IL-15 variant, was well tolerated while maintaining PD-1–dependent immune activity (Supplemental Figure 5D). Collectively, these data emphasize that cytokine attenuation enables safe, dual engagement of adaptive and innate cytotoxic immunity with PD-1–targeted IL-15 therapy. To further dissect the mechanism of action of the PD-1–targeted IL-15 mutein in tumor-bearing mice, we used the well-characterized B16-F10-OVA mouse melanoma model (Figure 5A). Anti–mPD-1–mutmIL-15 antitumor efficacy was tested against isotype control, anti–mPD-1, and an untargeted murine IL-15 mutein (mIgG–mutmIL-15). At the end of the study, tumors were harvested for multiomic characterization. Anti–mPD-1–mutmIL-15 induced the strongest antitumor efficacy with tumor weights (Figure 5B) and tumor sizes (Supplemental Figure 5E) being significantly reduced compared with the isotype control group at day 21 when mice were sacrificed and tissues collected for analyses. Consistent with the LCMV model showing preferential expansion and activation of antigen-specific T cells, IFN-γ production by splenocytes restimulated with OVA in vitro 11 days after a single dose of drug was significantly increased in mice treated with anti–mPD-1–mutmIL-15 (Figure 5B). IHC staining revealed significant reduction of PD-L1^+^ cells only within the tumors treated with anti–mPD-1–mutmIL-15 compared with the isotype control (Supplemental Figure 5F). Parallel IHC staining showed significant increase of CD8^+^ cells in all treated groups in comparison with isotype control, with anti–mPD-1–mutmIL-15 inducing the most substantial effect (Supplemental Figure 5F). We also noticed an upward trend in NKp46^+^ cells in the treated groups compared with the isotype control group, although variability in data show only significance for the mIgG–mutmIL-15–treated group (Supplemental Figure 5F). These data were supported by flow cytometry performed on dissociated tumors with significant increase in CD8^+^ T cells and CD49b^+^ NK cells (Supplemental Figure 5G). Given that NK cells do not express PD-1 but express IL-2 receptor, it is reasonable to consider that they might be equally stimulated by untargeted IL-15 as they would be by our PD-1–targeted IL-15 approach as previously shown in the literature (13). Additionally, we observed significant increase in CD4^+^ T cells as well as in conventional DCs (cDCs) in anti–mPD-1–mutmIL-15–treated tumors relative to isotype control, while FoxP3^+^ Tregs were not affected by treatment (Supplemental Figure 5G).

We further analyzed the treated tumors using the NanoString GeoMx Digital Spatial Profiler (DSP) platform with the mouse whole transcriptome assay. Antibodies against CD45 and CD8 were used as morphological markers to define immune cells, while a cocktail of antibodies against S100B/PMEL17 was used to mark melanoma cells (Figure 5D). Those markers, as well as parallel H&E images, were used to define 3 tumor compartments (“Leading-Edge”, “Tumor”, and “Necrosis”) and analyze over 300 regions of interest (ROIs). The abundances of 7 immune cell types were estimated using Microenvironment Cell Populations–counter (MCP-counter), including cytotoxic lymphocytes (CTLs) defined as the combination of T γδ cells, CD8^+^ T cells, and NK cells (14). For each compartment, the average estimated abundances of each cell type per tissue are compared against anti–mPD-1–mutmIL-15. Comparative analysis showed that anti–mPD-1–mutmIL-15 significantly increased the infiltration of CD8^+^ T cells and CTLs in both the leading-edge and tumor compartments (Figure 5C). Notably, CD8^+^ T cell infiltration in the leading edge was significantly greater with anti–mPD-1–mutmIL-15 than with anti–mPD-1 alone. Furthermore, treatment with anti–mPD-1–mutmIL-15 led to a significant increase in NK cell abundance in the tumor compartment, along with other innate and adaptive immune populations (Figure 5C and Supplemental Figure 5H). In contrast, anti–mPD-1 only significantly increased CTL infiltration in both compartments, without a significant effect on NK or CD8^+^ T cells compared with the isotype control (data not shown). Additionally, leveraging Th1 upregulated genes identified in a recently published mouse CD4^+^ T cell atlas (15), we further examined the pathway activity of CD4^+^ T cell subtype, Th1. The gene set variation analysis (GSVA) (16) showed that anti–mPD-1–mutmIL-15 significantly increased the activity of Th1 pathway in both the leading edge and the tumor compartments relative to the isotype control. In contrast, anti–mPD-1 only showed significance in the leading edge with a lower amplitude than the PD-1–targeted IL-15 mutein (data not shown). The results from GSVA supports previous observation in the LCMV model, in which we noted a strong potentiation of the CD4 response with viral-specific IFN-γ–secreting CD4^+^ T cells driven by anti–mPD-1–mutmIL-15. Overall, our findings illustrate that spatial transcriptomic profiling enables us to decipher various immune cell populations and pinpoint their responses to treatment within tumors.

We then performed gene expression analysis by comparing each treatment group to the isotype control group per spatial compartment. Differential gene expression (DGE) analysis revealed significant upregulation of genes associated with the cytotoxicity and activation of T and NK cells such as *Gzma*, *Gzmb*, *Tnfrsf18*, and *Ncr1* by anti–mPD-1–mutmIL-15 in both tumor and leading-edge compartments compared with the isotype control group (Figure 5E), while anti–mPD-1 and mIgG–mutmIL-15 showed limited upregulation of the same genes (Supplemental Figure 5I). In concordance with the gene expression data, *Ncr1*, a marker of NK cell activation also known as NKp46, was confirmed by IHC to be upregulated in tumors treated with the PD-1–targeted IL-15 mutein relative to the isotype control (Supplemental Figure 5F). Ingenuity Pathway Analysis (IPA) predicts the anti–mPD-1–mutmIL-15 molecule induced more strongly various biological functions of T and NK cells including activation, cytotoxicity, maturation, and proliferation than isotype control (Table 2) or anti–mPD-1 alone (Supplemental Table 3). In addition, anti–mPD-1–mutmIL-15 highly upregulated genes associated with dendritic cell accumulation and the recruitment of effector T cells such as X*cl1*, *Ifng*, C*xcr3*, *Cxcl9*, and *Cxcl10* in both leading edge and tumor compartment in comparison with isotype control (Figure 5E). The recruitment of cDCs confirmed by flow cytometry (Supplemental Figure 5G) as well as the upregulation of various biological functions of dendritic cells revealed by IPA (Table 3) suggest a positive immune feedback loop as previously described in the literature (17).

Overall, these data show that PD-1–targeted IL-15 mutein promotes intratumoral infiltration of innate and adaptive immune cells such as NK and CD8^+^ T cells and enhances their cell killing activity while promoting a strong Th1 response with upregulation of genes such as *Ifng* which led to a more robust anti-tumor effect than that seen with anti–mPD-1 or mIgG–mutmIL-15.

### SAR’877 human candidate molecule exhibits potent antitumor activity in mouse model.

**Figure 6:**
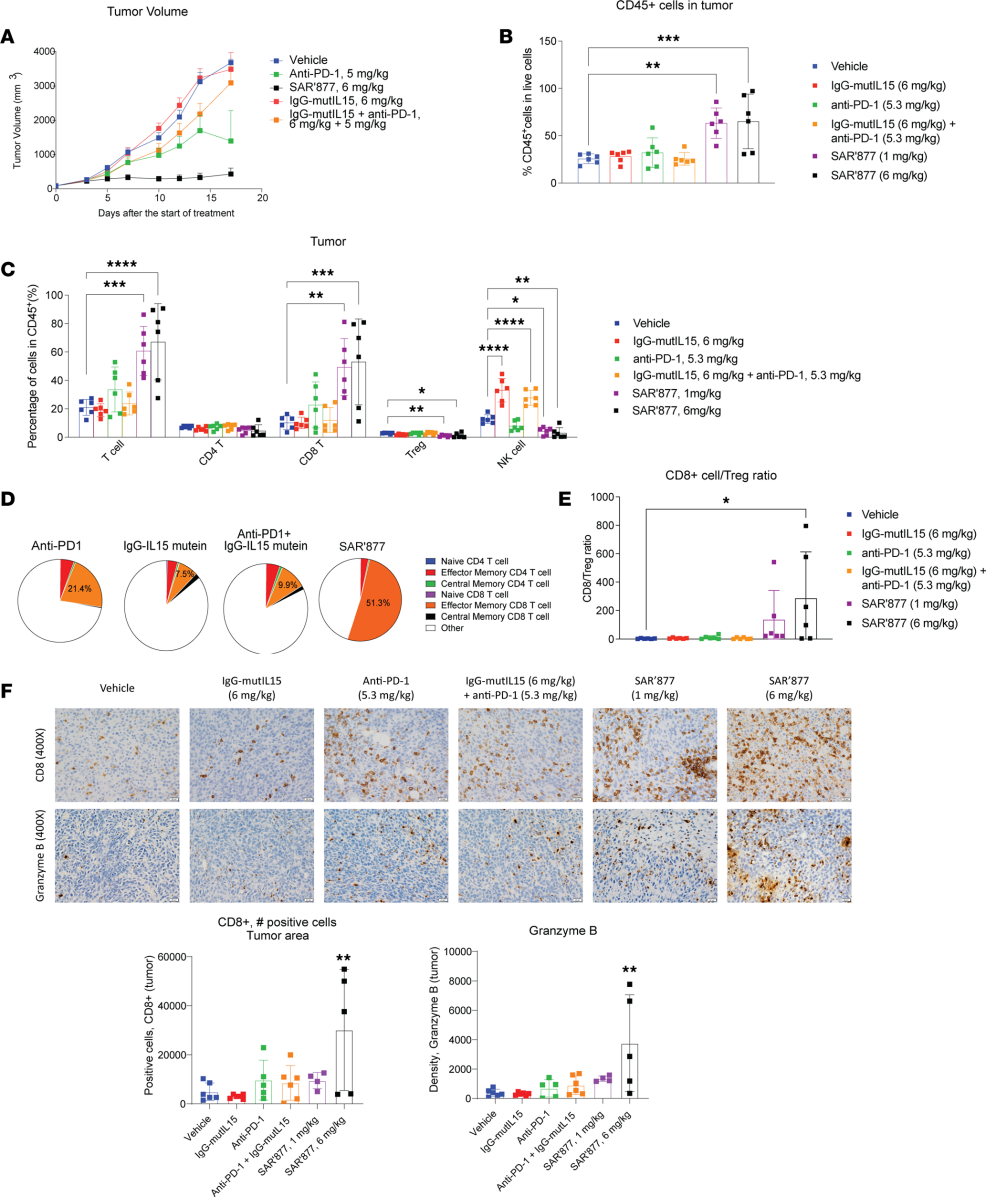
Antitumor efficacy of SAR’877 in a humanized mouse tumor model. SAR’877 was evaluated in BALB/c mice with human PD-1/PD-L1 knock-in bearing subcutaneous CT26-huPD-L1 tumors. (**A**) Tumor volume after IP treatment with SAR’877 or control antibody-cytokine fusions (anti–PD-1: BIW; all others: QW; *n* = 6/group). (**B**–**F**) Ex vivo tumor analyses: CD45^+^ cell frequency by flow cytometry (**B**), T and NK cell phenotyping (**C**), TIL subpopulation characterization (**D**), CD8^+^ T cell/Treg ratio (**E**), and CD8 and Granzyme B expression by IHC (**F**). Data are presented as bar plots showing mean ± SD. Statistical significance was assessed by 1-way ANOVA followed by Dunnett’s multiple comparisons test versus vehicle group. *P < 0.05; **P < 0.01; ***P < 0.001; ****P < 0.0001. Scale bar: 20 �m.

To test the human SAR’877 molecule, we utilized a mouse model in which BALB/c mice with knock-ins of human PD-1 and PD-L1 genes were engrafted with s.c. CT26 tumors expressing human PD-L1 (CT26-huPD-L1). A single i.v. dose of SAR’877 was administered to tumor-bearing mice, and tumor size was monitored. We initially tested a range of SAR’877 doses to gain insights into efficacy and tolerability profiles. All doses tested, ranging from 0.5 to 12 mg/kg, resulted in significant antitumor efficacy in a dose-dependent manner (Supplemental Figure 6A). Single-dose treatment was generally well tolerated, with no body weight loss observed at doses up to 6 mg/kg, while SAR’877 administered at 12 mg/kg induced a transient and reversible body weight loss of ~5% (Supplemental Figure 6A). In further experiments, we dosed mice once weekly for 3 weeks and observed SAR’877 induced strong inhibition in tumor growth resulting in tumor stasis at 6 mg/kg (Figure 6A). Among the treated mice, 2 individual mice treated with SAR’877 exhibited complete tumor clearance with no regrowth when monitored up to 87 days. Treatment with nontargeted IgG–mutIL-15 in combination with anti–PD-1 was less efficacious compared with SAR’877, inducing only a minor delay in tumor growth (Figure 6A). Similar results were observed when higher doses were tested, with 12 mg/kg SAR’877 showing stronger antitumor efficacy compared with the combination of anti–PD-1 + IgG–IL-15 mutein (Supplemental Figure 6, B and C). These results demonstrate the superior activity of SAR’877 compared with combination of anti–PD-1 and cytokine as separate agents. To further probe the role of PD-1 targeting on SAR’877 activity, SAR’877 was coadministered with anti–PD-1 antibody, which would compete for PD-1 binding and interrupt the PD-1–targeted activity of SAR’877. Coadministration of anti–PD-1 with SAR’877 resulted in reduced tumor growth inhibition (TGI = 68.2%) compared with SAR’877 (TGI=94.8%) (Supplemental Figure 6C).

We utilized the same mouse tumor model to investigate the mechanism of action of the SAR’877 antibody-cytokine fusion. Human PD-1/PD-L1 knock-in mice bearing CT26-hPD-L1 tumors were treated with a single dose of SAR’877 or control treatments and tissues were collected 7 days later for analysis. Flow cytometry analysis revealed that SAR’877 at a dose of either 1 mg/kg or 6 mg/kg significantly increased the percentage of CD45^+^ cells in the tumor microenvironment, while anti–PD-1, IgG–mutIL-15, and the combination of anti–PD-1 and IgG–mutIL-15 had minimal to no effects (Figure 6B). Among CD45^+^ cells in the tumor, SAR’877 preferentially expanded CD8^+^ T cells, with no substantial effect on CD4^+^ T cells (Figure 6C). The percentage of Tregs and NK cells decreased, potentially resulting from the strong preferential expansion of CD8^+^ T cells (Figure 6C). Deeper analysis of T cell subsets revealed that SAR’877 induced a striking expansion of CD3^+^CD8^+^CD62L^–^CD44^+^ effector memory cells while other subsets were largely unchanged (Figure 6D). Consistent with the preferential expansion of CD8^+^ effector memory cells and limited effects on intratumoral CD4^+^ T cells, the CD8^+^ T cell/Treg ratio was strongly increased in tumors following SAR’877 treatment (Figure 6E). The increased T cell infiltration observed by flow cytometry was further confirmed by IHC analysis, which revealed significantly increased CD8^+^ positivity and increased granzyme B in the microenvironment of SAR’877-treated tumors (Figure 6F).

## Discussion

Following cancer and persistent viral infections, elevated antigen levels and inflammatory immune environment drive T cell exhaustion, impairing proper control of tumors and viral infections alike. The breakthrough discovery that antibody blockade of PD-1 could reinvigorate exhausted T cells led to a revolution in cancer treatment and markedly extended survival of patients with late-stage disease (10, 18, 19). Still, most patients with cancer do not respond to or experience disease relapse while on anti–PD-1 therapy, highlighting the urgent need to develop more effective therapeutic approaches. Given cytokines’ well-known abilities to drive immune function, they have been explored to overcome this barrier. Common γ chain (g_c_) cytokines like IL-2 and IL-15 generally act on lymphocytes over short distances through autocrine, paracrine, or trans presentation (20). As such, g_c_ cytokines are highly potent and short-lived molecules allowing robust local immunity while minimizing damaging systemic inflammation. These intrinsic qualities of g_c_ cytokines have necessitated engineering strategies to optimize their use as a systemically administered cancer immunotherapy. Strategies aimed at enhancing half-life include cytokine fusions to the Fc domain of antibodies and polymer conjugates like polyethylene glycol (PEG) or fusion to human albumin. To minimize the systemic toxicity from cytokine therapeutics, conditional activation through tumor protease cleavable masking, pH-dependent binding, and allosteric modulation are all being explored (5).

The immunocytokine, which conjugates a specific monoclonal antibody to an immune activating cytokine, is another strategy that has gained growing interest over the past several years (21). For lymphocyte stimulating g_c_ cytokines like IL-2 and IL-15, immunocytokines have been developed that target either the tumor or immune cells. The former strategy posits that cytokines delivered at the cancer site can activate effector immune cells in the tumor microenvironment (trans activation), whereas the latter attempts to deliver cytokine to specific immune cells (cis activation) in the periphery or tissues in hopes of potentiating antitumor immunity. Both approaches intend to minimize dose-limiting toxicities that come with systemic administration while focusing cytokine signaling to specific environments or cell types to optimize anti-tumor responses (21). Here, we explored the immunocytokine SAR’877, composed of a high-affinity PD-1 antibody with a detuned IL-15/IL-15Rα sushi complex, to preferentially deliver IL-15 stimulation to PD-1^+^ T cells over any peripheral immune cell expressing the IL-2Rb and g chains. Whereas relatively low doses (μg/kg) of WT IL-15/IL-15Rα has been associated with toxicity in mice, including body weight loss (22), mice treated at significantly higher doses (mg/kg) with a murine surrogate of SAR’877 (anti–mPD-1–mutmIL-15) showed antitumor activity across several syngeneic tumor models in absence of body weight loss.

Using the PD-1–targeted IL-15 mutein, we have demonstrated superior rescue of T cell function in both mouse and human exhausted CD8^+^ T cells. Importantly, the restoration of function was superior to anti–PD-1, mIgG–mutmIL-15, or mIgG–mutmIL-15 + anti–PD-1 treatment, indicating that targeting the cytokine to PD-1^+^ cells can provide added value over coadministration of anti–PD-1 + IL-15. This was true both in vitro and in vivo, using both syngeneic tumor and persistent viral infection models, in which we observed significant increases in CD8^+^ T cell responses with mouse and human anti–PD-1–mutIL-15 molecule. Notably, we observed a discrepancy with modulation of NK cell responses between syngeneic tumor and viral models, in which significant increases in NK cell numbers were observed in tumor models while no increase was observed in persistent LCMV infection following anti–mPD-1–mutmIL-15 treatment. This could be explained by differences in PD-1 expression on NK cells in the models. In tumors, infiltrating NK cells express moderate to elevated PD-1 on their surface while in LCMV Clone-13 infection, PD-1 expression has been reported to be low (23–25). Therefore, modulation of NK cell function with an anti–mPD-1–mutmIL-15 immunocytokine will likely depend on the model system under investigation.

While the effects on CD8^+^ T cells and NK cells with SAR’877 were expected, the magnitude of the effects on CD4^+^ T cell responses was more surprising. In the LCMV Clone-13 system, CD4^+^ T cells also become exhausted and can express PD-1 on their surface (26, 27); thus, it is possible that the SAR’877 mouse homolog would bind PD-1 on activated CD4^+^ T cells. Moreover, IL-15 has been shown to promote effector CD4^+^ T cell responses when presented in trans (28). In this study, we demonstrate that our anti–mPD-1–mutmIL-15 (SAR’877 mouse surrogate) promoted expansion of total numbers of IFN-γ–producing GP_67-77_ CD4^+^ T cells as compared with anti–mPD-1, mIgG–mutmIL-15, or mIgG–mutmIL-15 + anti–PD-1 treatment. Closer analysis of the CD4^+^ T cell population by scRNA-seq identified that both the anti–mPD-1–mutmIL-15 increased the frequencies of cycling CD4^+^ T cells and reduced clusters of terminally differentiated/exhausted CD4^+^ T cells (Supplemental Figure 4, D and E). The importance of these effects is buttressed by the fact that the enhanced control of clone-13 infection and elevated numbers and function of CD8^+^ T cells was completely abrogated in CD4 depleted animals. Similar enhancement of CD4^+^ T cell responses was also observed in the syngeneic B16-F10-OVA tumor model, in which treatment with anti–mPD-1–mutmIL-15 promoted better Th1 responses compared with anti–mPD-1 or mIgG–mutmIL-15 treatments and also correlated with better tumor control. However, enhanced CD4^+^ T cell numbers were not observed in CT26 tumors with human PD-1/PD-L1 knock-in mice/tumors. Whether this discrepancy is due to the specific tumor model used or some other parameter of the model is unclear and will require further investigation. Nevertheless, CD4^+^ T cell responses have been reported to promote checkpoint-mediated tumor control (29–32). Specifically, Th1-skewed CD4^+^ T cell responses, characterized by high IFN-γ production, have been associated with improved tumor control following immunotherapy, suggesting that enhancing Th1-like activity in CD4^+^ cells could synergize with checkpoint inhibitors (33).

Our study has some limitations. While the murine surrogate of SAR’877 should be more pharmacologically relevant for studying the compound in mice, we cannot ignore inherent species differences in cytokine-related toxicities and biodistribution differences related to species-specific patterns of IL-2 receptor β and γ chains. Likewise, the surrogate showed antitumor activity across a range of syngeneic tumor models, but it’s important to consider that the tumor-bearing mice are young, have relatively healthy immune systems, and have not been exposed to prior chemotherapeutic regimens, ICB, or other anticancer therapies.

SAR’877 is currently undergoing evaluation for safety and efficacy in patients with cancer (NCT05584670). Early clinical data has demonstrated a tolerable safety profile as well as antitumor activity in patients with solid tumors displaying primary and acquired resistance to ICB (34). Thus, our preclinical findings have begun to show translational validation in patients with cancer and warrant larger trials to determine the potential of SAR’877 to generate an antitumor immune response with less toxicity than first-generation cytokine therapies.

## Methods

### Sex as a biological variable

All mice used for B16-OVA tumor growth and spatial transcriptomics experiments were female to maintain consistency across studies. While only females were used, the findings are expected to be broadly applicable. Though additional studies in males could be performed to confirm generalizability. For other studies, both male and females were used.

### Cell lines

All cell lines were maintained in a humidified incubator at 37°C with 5% CO_2_. M07e human leukemia cells (DSMZ) were cultured in IMDM supplemented with 15% heat-inactivated FBS, with some experiments additionally including 20% 5637-conditioned medium. The hIL-2Rβ–HEK293 reporter cell line, generated in-house, was maintained in IMDM with 10% heat-inactivated FBS. Pan02 tumor cells were cultured in DMEM containing 10% heat-inactivated FBS, 100 U/mL penicillin, and 100 μg/mL streptomycin. hPD-L1/CT26 tumor cells were grown in RPMI-1640 supplemented with 10% heat-inactivated FBS, 100 U/mL penicillin, and 100 μg/mL streptomycin.

### Human immune cell proliferation

Human PBMCs were thawed and cultured in complete IMDM medium (Thermo Fisher Scientific, #12440053) with 10% heat-inactivated FBS (#16140071) at 1 × 10^5^ cells/well in 96-well plates with serial antibody dilutions. After 6 days at 37°C, proliferation was quantified using CellTiter-Glo Luminescent Cell Viability Assay (Promega, #G7570). For T cell phenotyping, cells were stained with fixable viability dye eFluor 780 (Invitrogen, #65-0865-14) for 10 minutes at 4°C, before being fixed and permeabilized using Foxp3/Transcription Factor Staining Buffer Set (Thermo Fisher Scientific, #00-5523-00). Cells were stained with fluorochrome-labeled antibodies against CD4 (clone OKT4, #317408), CD8 (clone SK1, #344710), CD56 (clone 5.1H11, #362552), Foxp3 (clone 150D, #320014), and Ki-67 (clone Ki-67, #350504) (all BioLegend) for 1 hour at 4°C. Proliferating T cells (Ki-67^+^) were analyzed by flow cytometry (Guava EasyCyte, Luminex, #4700-0020).

### Cis-targeting assay in human T cells

T cells were isolated from human PBMCs by negative selection using the EasySep Human T Cell Isolation Kit (STEMCELL Technologies). Cells were stimulated overnight with Dynabeads Human T-Activator CD3/CD28 (Thermo Fisher Scientific, #11131D), after which beads were removed and T cells were cultured for an additional 2 days. Activated T cells (1.25 × 10^5^ per well) were treated with immunocytokines for 15 minutes at 37°C, immediately fixed, and stained for flow cytometric detection of pSTAT5 using the Transcription Factor Phospho Buffer Set (BD Biosciences, # 563239).

### Cytokine release assay

Cytokine secretion was assessed by incubating human whole blood with vehicle, human recombinant IL-15 (500 nM, 0.1 molar equivalent of SAR’877) or SAR’877 (1 mg/mL) for 48 hours. Cytokine levels in the supernatants were quantified using a Luminex xMAP MAGPIX system.

### Human CD4 and CD8 antigen recall assay

PBMCs, obtained either as frozen vials from Cellular Technology Limited (CTL, #CTL-CP1) or from an internal bank originally sourced as fresh leukopaks from STEMCELL Technologies (#70500.1), were thawed at 37°C in RPMI-1640 medium (Thermo Fisher Scientific, #22400089). The median post-thaw viability was 93%. Freshly isolated PBMCs (2 × 10^5^ per well) were plated in 200 μL CTS AIM-V medium (Thermo Fisher Scientific, #0870112DK) with test compounds, with or without CD4 antigen (CPI positive control, ImmunoSpot, #CTL-CPI-005) or CD8 antigen (HLA-A2 CE peptide pool, ImmunoSpot, #CTL-CEF-004), in 96-well polypropylene round-bottom plates (Corning, #3798). Cells were stimulated with 9 serial concentrations of SAR’877, starting at 300 nM, and incubated for 5 days at 37°C with 5% CO_2_ before supernatant collection.

IFN-γ levels were measured using the MSD IFN-γ MULTI-ASSAY kit (MSD, #L451QOA-1) according to the manufacturer’s instructions and read on a MESO SECTOR S 600MM instrument. Data were analyzed using DISCOVERY WORKBENCH sof Tware (v4.0). Statistical analyses were performed using GraphPad Prism, and data are reported as mean ± SD.

### MIMIC CD8+ T cell exhaustion assay

Autologous CD8^+^ T cells were isolated from frozen PBMCs by negative magnetic selection (EasySep, STEMCELL Technologies). Antigen-presenting cells (APCs) were generated as previously described (35). CD8^+^ T cells were cocultured with autologous dendritic cells preprimed with antiviral, HLA-restricted peptides (BioSynthesis, Inc.) at a 60:1 ratio in X-VIVO 15 medium (Lonza, #02-060Q) at 37°C and 5% CO_2_ for 12–14 days, during which repeated antigen exposure induced T-cell exhaustion. Antigen-specific exhausted CD8^+^ T cells were identified using HLA Class I pentamers (ProImmune) and evaluated by flow cytometry.

After 12–14 days, exhausted CD8^+^ T cells were labeled with CFSE (Invitrogen, #C34554) and restimulated with fresh autologous APCs pulsed with the corresponding HLA Class I peptide. Cultures were treated with SAR’877 or control conditions and incubated for 7 days. Functional CD8^+^ T cells from the same donors served as peptide-stimulated controls. Proliferation was assessed by CFSE dilution and quantification of HLA-pentamer^+^ CD8^+^ T cells, reported as the total number of divided cells (cells undergoing ≥1 division), to best capture the magnitude of antigen-specific expansion.

Exhaustion markers were reassessed on antigen-specific populations by Flow Cytometry, and supernatants from the 7-day cultures were analyzed for IFN-γ and TNF-α using the Milliplex Human Cytokine 10-Plex kit (EMD Millipore). The following monoclonal antibodies were obtained from BD Biosciences (San Jose, CA): TIM-3 (clone 7D3; Cat. 565564), TIGIT (clone 741182; Cat. 747842), PD-1 (clone MIH4; Cat. 557860), and GITR (clone V27-580; Cat. 747661). LAG-3 (clone 3DS223H; Cat. 56-2239-42) was purchased from eBiosciences (Thermo Fisher Scientific). Cytokine measurements were acquired using Bio-Plex/Luminex instrumentation and analyzed with Bio-Plex Manager sof Tware (Bio-Rad).

Paired comparisons between treatment conditions were analyzed using the Wilcoxon signed-rank test, selected as the most appropriate nonparametric method for the sample size and within-donor study design.

### NK cell cytotoxicity

PBMCs were thawed at 37°C in RPMI-1640 medium (Thermo Fisher Scientific, #22400089). Post-thaw viability was 93%. NK cells were isolated by negative selection using the EasySep Human NK Cell Isolation Kit (STEMCELL, #17955). A total of 1.5 × 10^4^ NK cells were incubated with 5 × 10³ K562 target cells (ECCAC #89121407), which had been prelabeled using Cytolight Rapid Dye Red (Sartorius, #4706), in 200 μL of CTS AIM-V medium (Thermo Fisher Scientific, #0870112DK) within 96-well polypropylene round-bottom plates (Corning, #3798). Cells were stimulated with 6 serial compound concentrations starting at 300 nM. Target-cell growth was monitored for 5 days using an Incucyte system (2022B Rev2), and data were analyzed with Incucyte sof Tware (2022B Rev2). Statistical analyses were performed in GraphPad Prism (GraphPad, San Diego, CA); bar graphs show mean ± SD.

### Animals and housing conditions

Male or female mice aged 6–8 weeks were housed in temperaturecontrolled rooms (22°C ± 2°C) on a 12-hour light/dark cycle with free access to food and sterile water. Environmental parameters, including room temperature, relative humidity (55% ± 15%), and lighting schedules, were monitored and archived by laboratory animal sciences staff. Body weight and tumor volume were measured 2 to 3 times per week until study endpoints. Tumor volume was calculated as (length × width²)/2.

### Anti-tumor efficacy in 12 syngeneic tumor models (MuScreen)

BALB/c and C57BL/6J mice were inoculated s.c. with the designated numbers of tumor cells (all cell lines obtained from ATCC) in 0.1 mL PBS (Table 1), with day 0 defined as the day of inoculation. Tumor growth inhibition (TGI) was calculated as TGI% = (1 – Ti/Vi) × 100, where Ti and Vi represent the mean tumor volumes of the treatment and control groups, respectively (*n* = 10/group). All groups received a single dose of SAR’877 at 6 mg/kg, except CT26 and EMT6 cohorts, which were dosed at 12 mg/kg. Additional details for each syngeneic model are provided in Table 1. Mice were euthanized when individual tumors exceeded 3000 mm³ or when a group’s mean tumor volume surpassed 2000 mm³. Body weight was monitored throughout the study, and animals were euthanized if weight loss exceeded 20% relative to the first treatment day.

### Biomarker study in the B16-F10-OVA model

Female C57BL/6J mice were obtained from the Jackson Laboratory (Bar Harbor, ME). Although males were not included in these specific studies, the findings are expected not to be affected by sex. B16 F10 OVA cells (ATCC, #CRL-6475, modified by Sanofi to express OVA) were cultured in DMEM (Life Technologies, #11995065) supplemented with 10% heat inactivated FBS (#10082 147) and 7.5 μg/mL blasticidin (Gibco, #05756) at 37°C with 5% CO_2_. Cells were harvested using 0.25% trypsin EDTA (Life Technologies, #2520056) and resuspended in DPBS (Gibco, #14190144). On day 0, 120 mice were inoculated subcutaneously in the right flank with 0.5 × 10^6^ B16 F10 OVA cells. On day 9, animals were pooled and randomized into treatment and control groups (10 mice per group), with tumor volumes ranging from 46–148 mm³. Treatment began on day 10 (Supplemental Table 2). On day 21, tumors and spleens were harvested (Figure 5A). Tumors were weighed, halved, and processed either for formalin fixation (10% neutral buffered formalin for 24 hours followed by transfer to 70% ethanol and paraffin embedding for H&E, IHC, and GeoMx DSP analysis) or for enzymatic dissociation and flow cytometric profiling. Spleens were processed for splenocyte isolation and antigen restimulation assays.

### Assessing SAR’877 in CT26 model

hPD-L1-CT26 tumor cells (WuXi AppTec) were cultured in RPMI-1640 supplemented with 10% heat-inactivated FBS, 100 U/mL penicillin, and 100 μg/mL streptomycin. Human PD-1/PD-L1 transgenic BALB/c mice (GemPharmatech Co. Ltd.) were inoculated subcutaneously in the right flank with 0.5 × 10^6^ hPD-L1-CT26 cells in 0.1 mL PBS. In efficacy studies, treatment began on days 7–9 when mean tumor volumes were ~50–138 mm³; for the MoA study, dosing began on day 10 at ~173 mm³. Animals were assigned to groups using a blinded Excel-based stratified randomization tool based on tumor volume.

### Splenocytes isolation and antigen restimulation

Spleens were dissociated into single-cell suspensions by gently pressing the tissue through a 70 μm cell strainer (BD Falcon, #352350). Red blood cells were lysed using 10 mL of 1× RBC lysis solution (Miltenyi Biotec, #130-094-183). Cells were resuspended in complete RPMI-1640 medium (Gibco, #11875-085) supplemented with 10% FBS (#10082-1478), 1% Pen/Strep (#15140-122), 55 μM β-mercaptoethanol (#21985023), 1% NEAA (#11140-050), and 1 mM sodium pyruvate (#11360-070), before being seeded into 96-well plates (Costar, #3367). Cells were stimulated with 10 μg/mL OVA SIINFEKL peptide (AnaSpec, #AS-60193). After 2 days, IFN-γ levels in culture supernatants were quantified using the MSD mouse IFN-γ assay (Meso Scale Discovery, #K152QOD) and read on a Meso Sector S 600MM plate reader.

### IHC

Serial tumor sections were processed for IHC on the BOND RX autostainer platform. B16-F10-OVA tumors were stained with anti-CD8a (rat, eBioscience, clone 4SM15, #14-0808-82), anti-CD335/NKp46 (rat, BioLegend, clone 29A1.4, #137601), and anti-CD274/PD-L1 (rabbit, Cell Signaling Technology, clone E1L3N, #13684). CT26 tumors were stained with anti-CD8a (rabbit, Cell Signaling Technology, clone D4W2Z, #98941) and anti-Granzyme B (rabbit, Cell Signaling Technology, clone E5V2L, #44153). Quantitative image analysis was performed using the HALO® platform (Indica Labs) with the Cytonuclear v2 algorithm. Immunopositive cells were quantified within viable tumor regions, with necrotic areas excluded using a Random Forest–based classifier distinguishing “Tumor” from “Necrosis”.

### Preparation of single-cell suspension

Tumors were dissociated using the Tumor Dissociation Kit, mouse (Miltenyi Biotec, #130-096-730) according to the manufacturer’s instructions. Briefly, tumor halves were minced and enzymatically dissociated using the gentleMACS Octo Dissociator with heaters (Miltenyi Biotec, #130-095-937). Cell suspensions were resuspended and passed through a 70 μm MACS SmartStrainer (Miltenyi Biotec, #130-098-462). Cells were collected by centrifugation (at 300 g) and processed for downstream flow cytometry analysis.

### Flow cytometry analysis

Dissociated tumor cells were stained with Live/Dead dye (BioLegend, #423106), washed, and incubated with Fc block (BioLegend, #101319) for 5 minutes. Cells were then stained with surface antibodies against CD45 (BD, #564279), CD3 (BD, #561798), CD4 (BD, #564667), CD8 (BD, #612759), CD274 (BD, #568361), CD279 (BD, #748242), CD49b (BD, #562453), Gr-1/Ly6G (BD, #751415), CD11c (BD, #746392), CD69 (BD, #740460), CD317 (BD, #747604), CD11b (BD, #740861), CD103 (BioLegend, #121447), TCRγδ (BioLegend, #118124), H-2K^b^ (BioLegend, #116517), and I-A/I-E (BioLegend, #107622) for 20–30 minutes at 4°C. After washing, cells were fixed and permeabilized (eBioscience, #00-5523-00) for 30 minutes and stained intracellularly with anti-Foxp3 (BioLegend, #126419). Following a final wash, samples were resuspended in staining buffer containing CountBright beads (Invitrogen, #C36950). B16-F10-OVA tumors were acquired on a Cytek Aurora and analyzed with OMIQ; CT26 tumors were acquired on a BD Fortessa X20 and analyzed with FlowJo v10.

### scRNA-seq and analysis

Mouse splenic T cells (CD3^+^ CD19^–^CD11b^–^) isolated using flow cytometry, stained with DNA-barcoded hashtag TotalSeq-B antibodies (Biolegend) and subjected to single-cell transcriptome library preparation using the 3’ v3.1 transcriptome kit (10X Genomics). Libraries were sequenced using the recommended cycle settings and subjected to quality control, cell identification, read alignment and counting using cellranger (10X Genomics). Demultiplexing, normalization, dimensionality reduction, clustering, marker analysis were performed using Seurat v5 (35). RNA velocity analyses were peformed using velocyto (36), scvelo (37), and cellrank (38). Visualization of heatmaps utilized the ComplexHeatmap R package. Partition-based graph abstraction analysis (PAGA) was calculated using the cellrank implementation of PAGA (39).

### GeoMx DSP, spatial transcriptomics assay

Slide preparation: FFPE tumor sections (5 μm; *n* = 20 tumors, 5 per group) selected based on necrosis and IHC representation were mounted on Apex BOND superior adhesive slides (Leica Biosystems, #3800040), baked at 60°C for 1 hour, and stored at 4°C with desiccant. Within 2 weeks, slides were processed using the NanoString GeoMx DSP Automated RNA Slide Preparation protocol (MAN-10151-04) on a Leica BOND RX. Epitope retrieval was performed with BOND ER2 (Leica, #AR9640; 100°C, 10 min) followed by proteinase K digestion (1 μg/mL, 15 min). Sections were stained with the GeoMx Melanoma TME Morphology Kit (NanoString, #GMX-PRO-MORPH-MMEL-12) and AF647 anti-CD8α (Abcam, clone EPR21769, #ab237365). In situ hybridization was performed with the GeoMx Mouse Whole Transcriptome Atlas probe mix (>20,000 genes), each probe containing a photocleavable oligo tag.

ROI selection and collection: Slides were imaged on the GeoMx DSP instrument. Three compartments (Leading Edge, Tumor, Necrosis) were defined per tumor using morphological markers and H&E. Eight circular ROIs (250 μm diameter) per compartment were collected (24 per tumor; 23 for isotype controls), strictly within tumor boundaries. Oligonucleotide tags were released by UV exposure and collected into 96-well plates.

Library preparation, sequencing and GeoMx mapping: Libraries were prepared following the GeoMx DSP NGS readout protocol (MAN-10153-02). Library quality was assessed using the Agilent 4200 TapeStation with D1000 ScreenTape (Agilent, #5067-5582), and concentrations measured using the Qubit Flex Fluorometer and 1X dsDNA BR Assay Kit (ThermoFisher, #Q33262). Sequencing was conducted on an Illumina NovaSeq 6000 (v1.5 reagents, 27 bp paired reads, i5 reverse orientation). BCL files were converted to FASTQ and processed via the GeoMx NGS Pipeline v1.2 to generate DCC files, which were mapped back to tissue to produce spatial expression profiles. A total of 474 ROIs were successfully sequenced and spatially mapped.

### GeoMx DSP data analysis

#### Data post-processing.

We collected 474 ROIs from 20 tumors (5 tumors per group). After QC filtering (minimum nuclei ≥ 100, minimum negative probe count ≥ 10), 146 ROIs were removed. Gene-level QC using standR (addPerROIQC; min_count = 10, sample_fraction = 0.9) retained all genes. Gene expression was normalized using Trimmed Mean of M values (TMM) to reduce technical variation across ROIs.

#### Cell-type deconvolution analysis.

Immune cell abundances (B cells, T cells, CD8 T cells, cytotoxic lymphocytes, NK cells, monocytic lineage cells, neutrophils) were estimated per ROI using MCP-counter. Mean compartment-level abundances were compared across samples. Th1-associated genes were used to compute Th1 signature scores via ssGSEA (GSVA). Statistical comparisons for cell-type abundances and Th1 scores were performed using Wilcoxon tests with FDR correction.

#### DEG and IPA.

DEG analysis was conducted with edgeR (v4.0.0). Genes with FDR < 0.05 and |logFC| > 1 were considered significant. These DEGs were subjected to IPA disease and function analysis.

### Statistics

Statistical analyses were conducted using GraphPad Prism (GraphPad, San Diego, CA). Scatter and box plot data are presented as mean ± SD. Normality of residuals was assessed by 1-way ANOVA diagnostics, including Q–Q plot inspection and normality tests. For tumor weight and IHC analyses, variance homogeneity was evaluated using residual plots and the Brown–Forsythe test. When assumptions of normality and equal variances were met, group means were compared with the isotype control using Dunnett’s multiple comparisons test. If normality was not achieved, data were log-transformed and reanalyzed using the same workflow, with results back-transformed to the original scale. For flow cytometry, when the 2-way ANOVA F-statistic was significant, post hoc comparisons were performed using either the Games–Howell test (unequal variances) or Tukey’s test (equal variances). *P* values of less than 0.05 were considered significant.

### Study approval

All animal studies were performed following the guidance and approval by the IACUC at Sanofi (United States) and conducted in accordance with AAALAC International guidelines.

The use of deidentified human PBMCs, whole blood, and isolated CD4^+^ and CD8^+^ T cells from commercial vendors (Cellular Technology Limited, STEMCELL Technologies) was determined to be exempt from IRB review under 45 CFR 46.104(d) (4). This exemption applies to all human cell-based assays described in this study.

### Data availability

The datasets generated or analyzed in this study are available in the Supporting data values. The scRNA-seq data have been deposited in GEO under accession no. GSE323389, and the spatial transcriptomics data under accession no. GSE324814.

## Author contributions

Conceptualization was contributed by DL, JP, AH, VRF, DRS, and JRT. Methodology was contributed by JZ, AMB, VCR, DSB, and XL. Investigation was contributed by IP, M. Bernardo, JT, JZ, KLM, JSL, MGD, AC, RC, DL, M Bismal, SM, YZ, IML, and VCR. Formal analysi was contributed by: IP, JZ, JSL, AMB, and RC. Funding acquisition was contributed by JP, DRS, VRF, and JRT. Supervision was contributed by JP, AH, XL, VRF, DRS, and JRT. Writing of the original draft was contributed by IP, M Bernardo, JT, JSL, JZ, DRS, and JRT. DRS and JRT assigned co–first authorship to IP, M Bernardo, and JT with the co–first author order determined based on the relative duration of each author’s involvement with the project.

## Conflict of interest

MB, JT, JSL, AC, AMB, MGD, RC, YZ, IML, VCR, DSB, AH, XL, VRF, and DRS were employees of Sanofi in the course of this work and may hold stock in Sanofi. DL, SM, and JP were employees of Kadmon Corporation, which was acquired by Sanofi, in the course of this work. JRT received a research grant from Sanofi.

## Funding support

This work is, in part, the result of NIH funding and is subject to the NIH Public Access Policy. Through acceptance of this federal funding, the NIH has been given a right to make the work publicly available in PubMed Central.

- Sanofi
- Kadmon Corporation, a Sanofi research grant (to JRT)
- NIH under grant 5R01AI164744
- NIH (K22CA292568; to JZ)
- University of Utah and the Huntsman Cancer Foundation

## Supplementary Material

Supplemental data

Supporting data values

## Figures and Tables

**Table 1:**
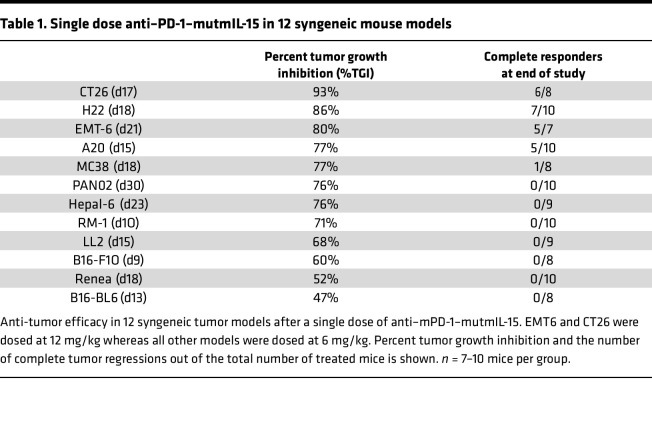
Single dose anti–PD-1–mutmIL-15 in 12 syngeneic mouse models

**Table 2:**
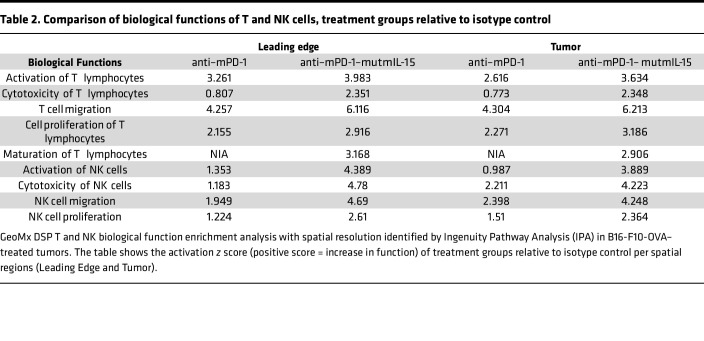
Comparison of biological functions of T and NK cells, treatment groups relative to isotype control

**Table 3:**
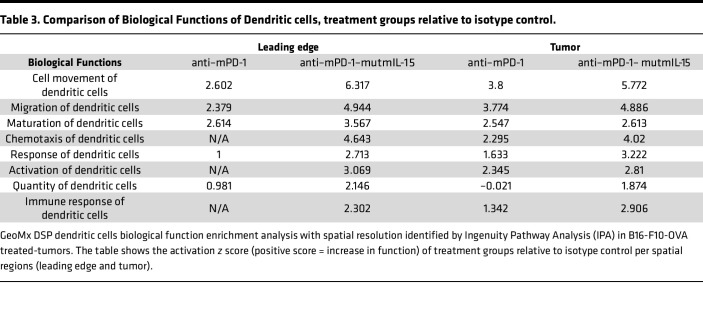
Comparison of Biological Functions of Dendritic cells, treatment groups relative to isotype control.

## Version 1 04/30/2026

In-Press Preview

## Version 2 06/22/2026

Electronic publication
