# Supplementary material for: PD-1–targeted IL-15 mutein activates CD8+ and CD4+ T cells in infection and cancer
Source: JCI Insight. 2026 Apr 30;11(12):e198701. doi: 10.1172/jci.insight.198701 (PMC13313502; doi:10.1172/jci.insight.198701)
Supplement: Supplemental data [file jciinsight-11-198701-s241.pdf]

SUPPLEMENTARY MATERIAL AND METHODS

M07e proliferation

M07e (DSMZ, #ACC 104) and PD-1^+ M07e cells (Sanofi) (2.5×10^4/well) were seeded in IMDM with 4% heat-inactivated FBS and incubated with serially diluted test antibodies for 3 days (M07e) or 4 days (M07e-PD-1). Proliferation was quantified using the CellTiter-Glo Luminescent Cell Viability Assay (Promega, #G7570).

HEK binding

HEK293 cells expressing hIL-2Rβ or hPD-1/hIL-2Rβ (Sanofi) were incubated with test antibodies (1 hour at 4°C or 3 hours at room temperature), washed, and stained with PE-labeled goat anti-human IgG/Fc (Jackson ImmunoResearch, Polyclonal, #109-036-098) for 30 minutes at 4°C. Binding was quantified by flow cytometry on a Guava EasyCyte (Millipore).

C1q binding ELISA

ELISA Plates were coated with test articles overnight at 4°C, blocked with PBS-T/BSA, and incubated with serial dilutions of C1q protein (Sigma, #C1740-1MG) for 1 hour at room temperature. Bound C1q was detected using TMB peroxidase substrate (SeraCare), and absorbance was measured on a PHERAstar FSX plate reader.

Complement dependent cytotoxicity assay

CHO-K1 cells expressing PD-1 (GenScript PROBIO) were incubated with antibody-cytokine fusions for 30 minutes at room temperature, followed by addition of normal human serum complement (NHSC, 20%) and incubation for 4 hours at 37°C. Cell viability was measured by CellTiter-Glo, and luminescence was quantified on a PHERAstar FSX plate reader. Percent lysis was calculated as: % lysis = 100 × [1 - ($RLU_{treated}$ - $RLU_{control}$)/($RLU_{untreated}$ - $RLU_{background}$)], where $RLU_{untreated}$ represents cells with NHSC only and $RLU_{background}$ represents media with NHSC alone.

Antibody-dependent cytotoxicity assay

CHO-K1 cells expressing PD-1 (GenScript PROBIO) were incubated with antibody-cytokine fusions for 30 minutes at room temperature. Human PBMC effector cells were added at a 50:1 ratio of effectors to targets, and cells incubated in cell culture conditions for 6 hours. Cell viability was assayed by LDH assay (Roche, #11644793001) and optimal density measured using PheraStar FSX plate reader. Target cell lysis was calculated according to the following equation: % lysis = 100 x ($OD_{treated}$ – $OD_{control}$)/(OD_{max} – OD_{min}), in which OD_{max} is the measurement of cells lysed with buffer and OD_{min} is the measurement of cells with no treatment.

pSTAT5 assay in mixed M07e and PD-1$^+$ M07e cells

M07e (DSMZ, #ACC 104) and PD-1$^+$ M07e cells (Sanofi) were stained with CellTrace Violet (Thermo Fisher Scientific, #C34571) and CellTrace Far Red (Thermo Fisher Scientific, #C34572), respectively. Cells (3.5 × 10^5/well) were treated with stimulating agents for 15 minutes at 37°C, fixed, and stained for pSTAT5 (BD Biosciences, clone 47/Stat5(pY694), #612598) using the BD Transcription Factor Phospho Buffer Set (BD Biosciences, #563239). Flow cytometry used

CellTrace dyes to distinguish cell populations, and pSTAT5 mean fluorescence intensity was normalized to the minimum value for each cell line to calculate fold change.

Single cell sequencing analysis

Gene expression was analyzed on tumor-infiltrating immune cells isolated from human tumor samples. Published single-cell sequencing data from cells derived from lung, ovarian, colorectal, breast and esophageal tumors from the following publications were analyzed using BioTuring (40-44). BioTuring Talk2Data tool (8) was utilized for analysis. Data for 1,182,854 cells was merged and expression of PD-1, IL2Rβ and IL2Rγ was analyzed across immune cell subsets. Immune cell populations were matched across studies and automatically characterized by BioTuring Inc. based upon the reference.

***In vitro* mouse T cell exhaustion assay**

An assay using a YFP–IFN-γ reporter strain was developed and scaled to high throughput format as described previously (11). Briefly, at day 15 post-infection with LCMV Cl13, B cells were depleted from splenocytes of FN-γ–YFP mice. Next, 2×10^5cells were seeded onto 96-well plate in complete T cell media supplemented with 2 µg/ml LCMV-specific CD8 peptides (GP33-41, NP396-404 and GP276-286) and 5 µg/ml CD4 peptide (GP61-80) in the presence of anti-mPD-1-mutmIL15 or controls. After 5 days of culture, cells were stained with Ghost Dye™ Violet 510 (Cytek, #13-0827-T100) to exclude dead cells, followed by surface staining with the following antibodies: anti-CD45 (BD, #564279), anti-CD4 (BD, #564667), anti-CD8 (BD, #612759), anti-

CD3 (BD, #561798), and anti-CD44 (BD, #564109). Flow cytometry analysis was performed to identify live IFN-γ^+ CD44$^+$ CD8$^+$ T cells.

Ex vivo mouse T cell stimulation assay

Splenocytes (2×10^6) were stimulated in complete T cell medium with the LCMV-derived peptides GP33 (2 µg/mL) or GP61 (5 µg/mL) for 1 hour at 37°C, after which Brefeldin A was added for an additional 5 hours. Cells were then labeled with Ghost Dye Violet 510 (Cytek #SKU13-0827-T100) and stained for surface markers using anti-CD45 (BD Biosciences, clone 30-F11, #564279), anti-CD4 (BD Biosciences, clone GK1.5, #564667), anti-CD8 (BD Biosciences, clone 53-6.7, #612759), and anti-CD3 (BD Biosciences, clone 17A2, #561798). Intracellular staining was subsequently performed with anti–IFN-γ (BioLegend, clone XMG1.2, #505841), anti–TNF-α (BD Biosciences, clone MP6-XT22, #557644), and anti–IL-2 (BioLegend, clone JES6-5H4, #503825) to quantify IFN-γ^+, TNF-α^+, and IL-2$^+$ CD8$^+$ T cells.

Virus growth and titration

LCMV Cl13 (45) was grown on BHK-21 cells (ATCC, # CCL-10) as previously reported(46). Viral titer was determined as previously reported (47). Briefly, frozen stock or serum was thawed and 10µL was used to perform 10-fold serial dilutions on VeroE6 cells (ATCC, # CRL-1586). Focus forming units were quantified using ImmunoSpot analyzer.

Mice and *in vivo* LCMV infection

Mouse studies were approved by the Institutional Animal Care and Use Committee (IACUC) of The Scripps Research Institute. C57BL/6 WT mice were obtained from an institutional breeding colony derived from Jackson breeders (Jackson #000664). All mice were bred and maintained under specific pathogen-free conditions.

Mice were intravenously infected with $2x10^6$ LCMV Cl13. Mice harboring chronic LCMV infection were treated with antibody-cytokine fusions and controls on day 20, 27, and 34 post-infections. Serum was collected on day 13, 27 and 35 post-infections to determine serum viral titer. Spleens were collected at day 35 post-infection for cellular analyses by flow cytometry.

SUPPLEMENTARY FIGURES

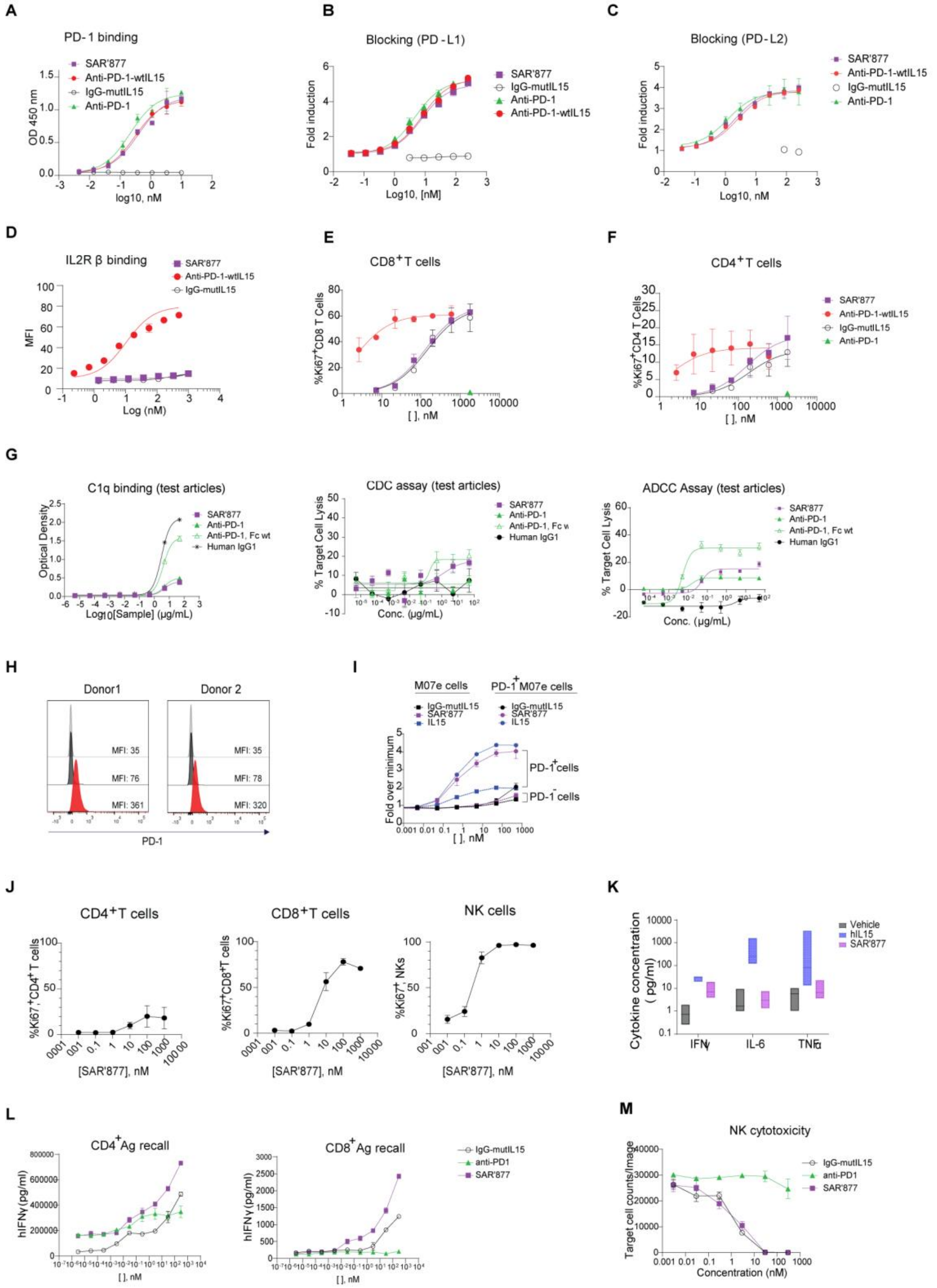

Fig. S1 ***In vitro*** **characterization of SAR'877 activity**

(A) Binding of SAR'877 and other antibody-cytokine fusions to plate bound human PD-1 protein as detected by ELISA. (B) Effects of antibody-cytokine fusions on PD-1 mediated immunosuppression as measured by Promega PD-L1 blocking reporter assay (C) and Promega PD-L2 blocking reporter assay. (D) Binding to HEKBlueIL2Rb cells detected by flow cytometry. (E) Proliferation of $CD8^+$ T cells and (F) $CD4^+$ T cells among PBMCs treated with SAR'877 *in vitro* for 6 days; proliferation was measured by flow cytometry of Ki-67^+ cells and the average of two PBMC donors is shown. (G) Binding of SAR'877 and other antibody-cytokine fusions to C1q protein as detected by binding ELISA, CDC activity against PD-1 expressing target cells measured by *in vitro* toxicity assay, ADCC activity against PD-1 expressing target cells measured by *in vitro* cytotoxicity assay. Anti-PD-1 antibody with an unmodified Fc region was included as a control. (H) PD-1 expression on total T cells following stimulation and resting was detected by flow cytometry. Top histogram (light gray) represents unstained cells; middle histogram (dark gray) represents unstimulated cells; bottom histogram (red) represents stimulated and rested cells. MFI is shown on each graph. Data is shown for the same human PBMC donors shown in figure 1E. (I) M07e and PD-1^+ M07e cells were mixed in a 1:1 ratio, incubated with SAR'877, IgG-mutIL15, or human recombinant IL15, and activation measured by flow cytometry of pSTAT5. Graphs show mean and standard deviation (n=3 replicates). (J) Proliferation of $CD4^+$ T cells, $CD8^+$ T cells, and NK cells among PBMCs treated with SAR'877 *in vitro* for 6 days; proliferation was measured by flow cytometry of Ki-67^+ cells. An average of two representative PBMC donors are shown. (K) Cytokine production in whole blood treated with human recombinant IL15 or SAR'877. n=3 donors. (L) PBMCs were stimulated with CD4 or CD8 peptide pools in the presence of antibody-cytokine fusions for 5 days and IFN-γ in the culture supernatants was measured (n=2 donors, 1 donor shown, technical triplicates). (M) Human NK cells were cocultured with K562 target cells

in the presence of SAR’877 for 5 days, and target cell viability was monitored. Mean and standard deviation (n=2 donors tested, 1 shown; technical triplicate).

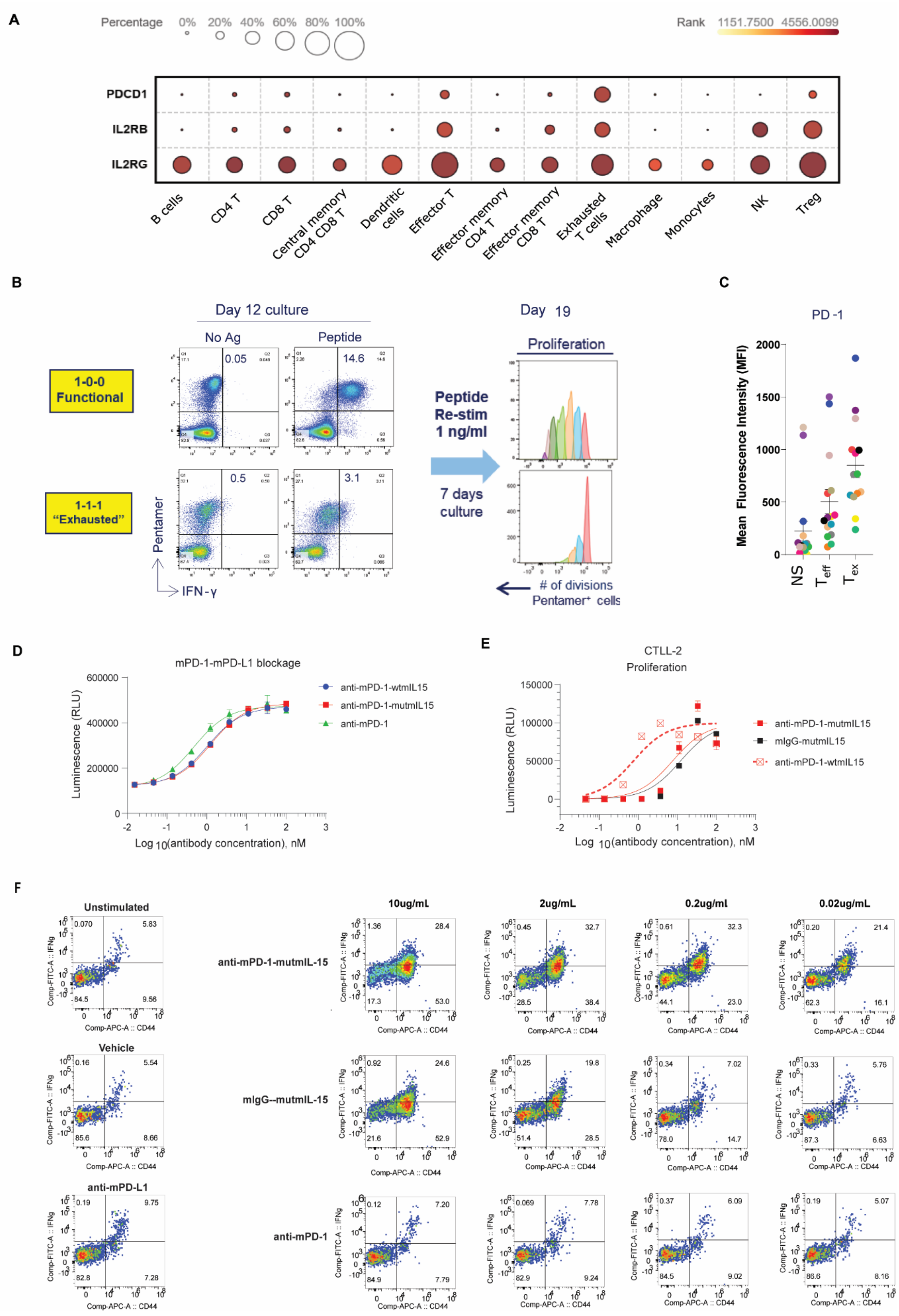
A
Percentage 0% 20% 40% 60% 80% 100%
Rank 1151.7500 4556.0099
PDCD1
IL2RB
IL2RG
B cells
CD4 T
CD8 T
Central memory CD4 CD8 T
Dendritic cells
Effector T
Effector memory CD4 T
Effector memory CD8 T
Exhausted T cells
Macrophage
Monocytes
NK
Treg
B
Day 12 culture
No Ag
Peptide
0.05
14.6
0.5
3.1
1-0-0 Functional
1-1-1 "Exhausted"
Pentamer
IFN-γ
Peptide Re-stim 1 ng/ml
7 days culture
Day 19
Proliferation
# of divisions
Pentamer⁺ cells
C
PD-1
Mean Fluorescence Intensity (MFI)
2000
1500
1000
500
0
NS
Teff
Tex
D
mPD-1-mPD-L1 blockage
Luminescence (RLU)
600000
400000
200000
0
anti-mPD-1-wtmIL15
anti-mPD-1-mutmIL15
anti-mPD-1
Log 10(antibody concentration), nM
E
CTLL-2
Proliferation
150000
100000
50000
0
anti-mPD-1-mutmIL15
mIgG-mutmIL15
anti-mPD-1-wtmIL15
F
Unstimulated
Vehicle
anti-mPD-L1
10ug/mL
2ug/mL
0.2ug/mL
0.02ug/mL
anti-mPD-1-mutmIL-15
mIgG--mutmIL-15
anti-mPD-1
Comp-FITC-A :: IFNg
Comp-APC-A :: CD44

124

G

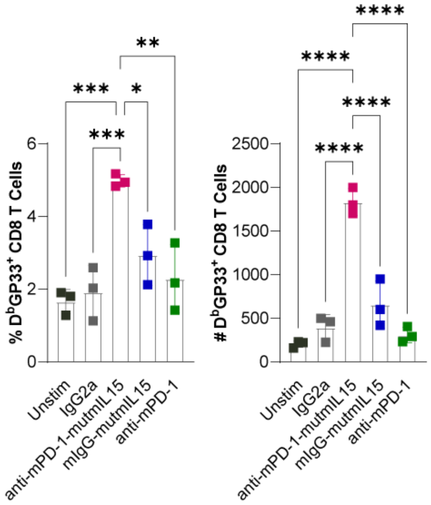
% D^bGP33^+ CD8 T Cells
# D^bGP33^+ CD8 T Cells
0
2
4
6
500
1000
1500
2000
2500
***
*
**
****
ns
Unstim
IgG2a
anti-mPD-1-mutmIL15
mIgG-mutmIL15
anti-mPD-1

H

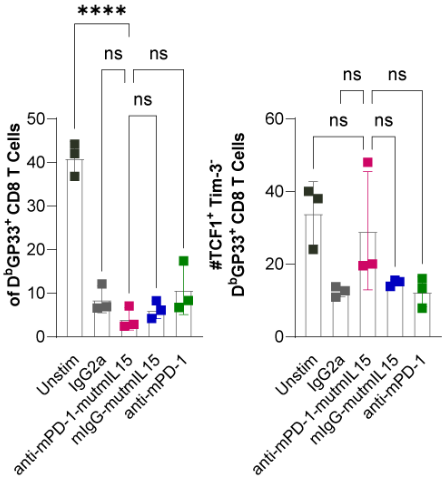
%TCF1^+ Tim-3^- of D^bGP33^+ CD8 T Cells
#TCF1^+ Tim-3^- D^bGP33^+ CD8 T Cells
0
10
20
30
40
50
60
****
ns
Unstim
IgG2a
anti-mPD-1-mutmIL15
mIgG-mutmIL15
anti-mPD-1

I

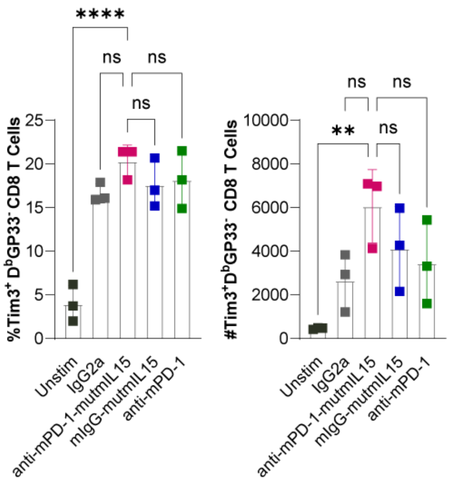
%Tim3^+ D^bGP33^- CD8 T Cells
#Tim3^+D^bGP33^- CD8 T Cells
0
5
10
15
20
25
2000
4000
6000
8000
10000
****
**
ns
Unstim
IgG2a
anti-mPD-1-mutmIL15
mIgG-mutmIL15
anti-mPD-1

J

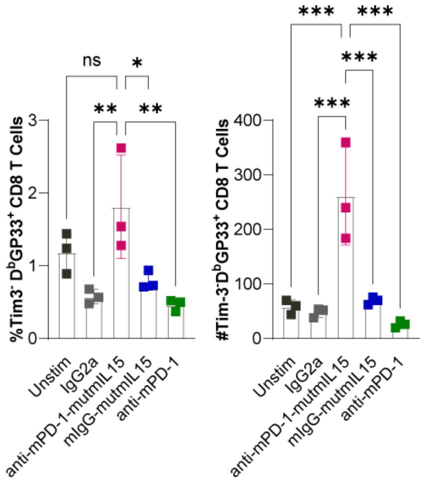
%Tim3^- D^bGP33^+ CD8 T Cells
#Tim-3^-D^bGP33^+ CD8 T Cells
0
1
2
3
100
200
300
400
ns
*
**
***
Unstim
IgG2a
anti-mPD-1-mutmIL15
mIgG-mutmIL15
anti-mPD-1

K

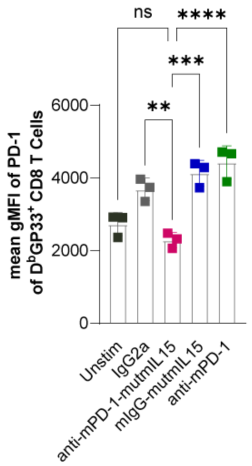
mean gMFI of PD-1 of D^bGP33^+ CD8 T Cells
0
2000
4000
6000
ns
****
***
**
Unstim
IgG2a
anti-mPD-1-mutmIL15
mIgG-mutmIL15
anti-mPD-1

125

Fig. S2 Human T cell exhaustion and characterization of SAR'877 mouse surrogate

(A) Expression of PD-1 (*PDCD1*), IL2Rβ (*IL2RB*) and IL2Rγ (*IL2RG*) by different subsets of tumor-infiltrating immune cells. Tumor-infiltrating cells isolated from lung, ovarian, colorectal, breast and esophageal tumors and analyzed by single-cell sequencing were pooled and analyzed using BioTuring Talk2Data tool. Data were derived from published datasets. (B) Antigen-specific CD8+ T cells from the MIMIC CD8+ T cell exhaustion assay, subjected to repeat peptide stimulation, show diminished IFN-γ production and reduced proliferation, indicative of an exhausted phenotype. (C) Exhausted T cells (T_{ex}) from the MIMIC $CD8^+$ T cell exhaustion assay exhibit high expression of PD-1 compared to non-exhausted controls (NS and T_{eff}). Surface PD-1 expression was assayed by flow cytometry. (D) Blockade of mouse PD-1 was measured using the Promega mouse PD-1 reporter assay. (E) Cytokine dependent mouse CTLL-2 cells were treated with antibody-cytokine fusions and proliferation was measured by CellTiter-Glo Assay. (F) Flow cytometry plots corresponding to Figure 2B depicting $CD44^+$ IFN-$\gamma^-$$YFP^+$ $CD8^+$ T cells five days after peptide stimulation. Frequency and total number of (G) D^bGP33^+CD8 T cells, (H) $TCF1^+Tim\text{-}3^-$of D^bGP33^+CD8 T cells, (I) Tim-$3^+$of D^bGP33^+CD8 T cells, (J) Tim-$3^-$of D^bGP33^+CD8 T cells, and (K) mean gMFI of PD-1 of D^bGP33^+CD8 T cells five days after peptide stimulation. Statistical significance was determined using one-way ANOVA followed by Dunnett's multiple comparisons test. * $P < 0.05$, ** $P < 0.01$, *** $P < 0.001$, **** $P < 0.0001$.

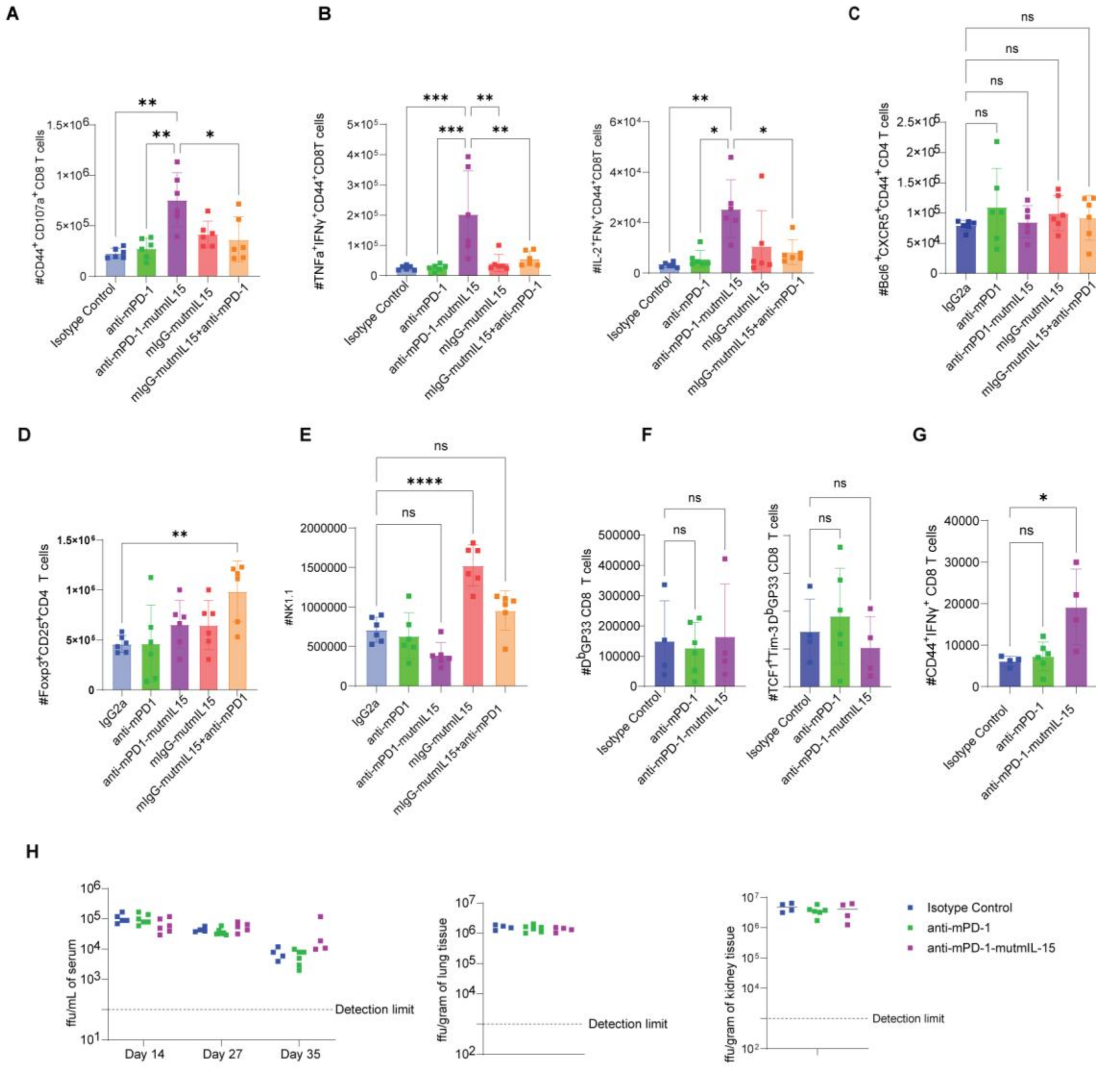

Fig. S3 SAR'877 surrogate and controls in LCMV infection model

Analyses of splenocytes of (A) CD107a, (B) IFN-γ^+TNF-α^+ and IFN-γ^+IL-2^+ expression on LCMV-specific CD8^+ T cells following *ex vivo* $GP_{33\text{–}41}$ peptide restimulation. (C) Total numbers of CXCR5^+Bcl6^+ T_{FH} cells. (D) total numbers of FoxP3^+ T regulatory T cells. (E) Total numbers of NK1.1^+ cells. (F & G) Following CD4^+ T cell depletion and chronic LCMV infection, virus-specific CD8^+ T cells were measured by flow cytometry in mice chronically infected with LCMV Cl13 that were treated with anti-mPD-1-mutmIL15, anti-mPD1, or IgG2a and Statistical

Internal

significance was determined using one-way ANOVA followed by Dunnett's multiple comparisons test. * $P < 0.05$, ** $P < 0.01$, *** $P < 0.001$, **** $P < 0.0001$. (H) viral titer in serum, lung and kidney were determined.

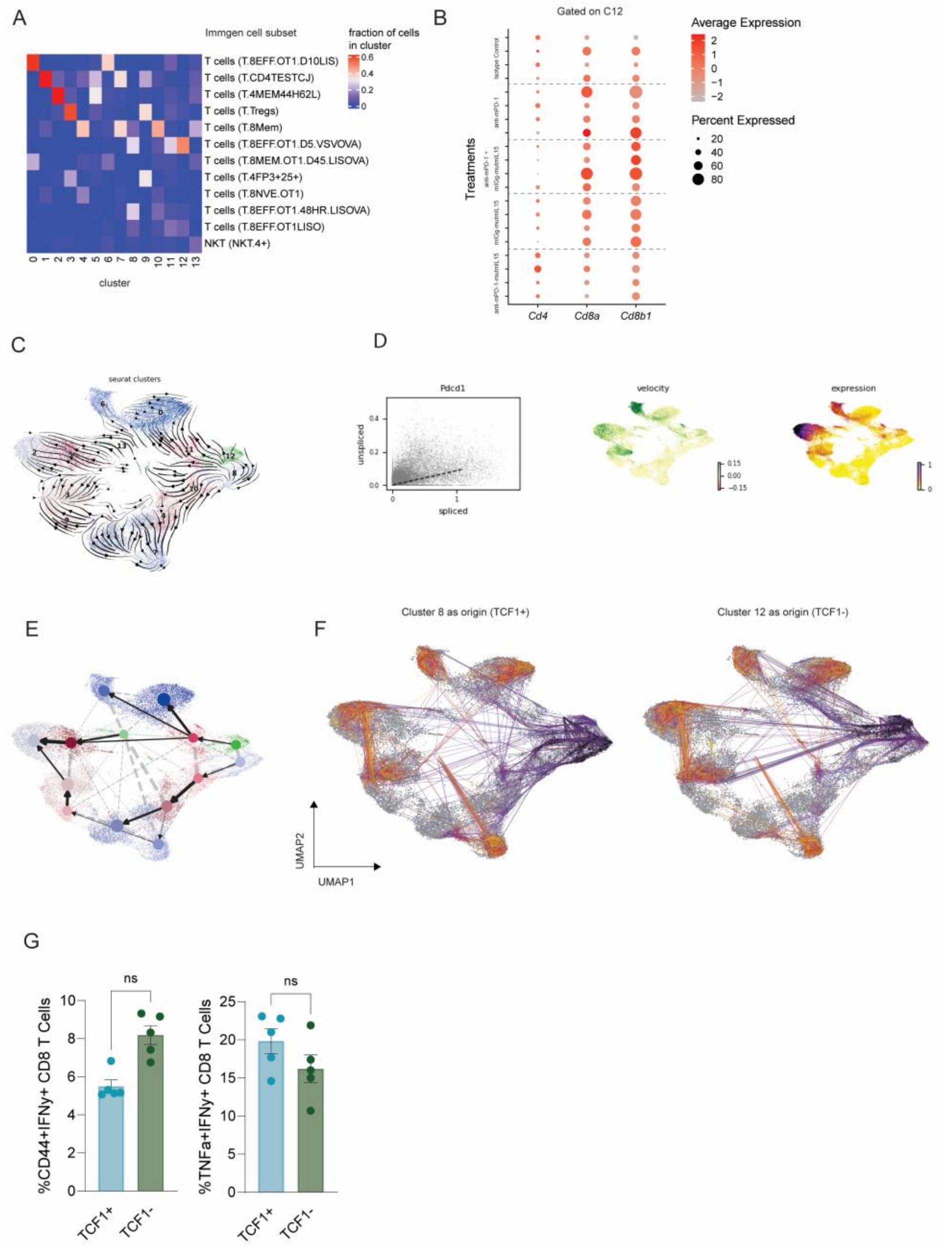

Fig. S4 LCMV infection model scSeq analyses

(A) Heatmap of the predicted fractions of each ImmGen cell subset per cluster as determined by SingleR using ImmGen RNA-seq data as reference (GSE109125). (B) Dotplot of the relative expression of *Cd4*, *Cd8a* and *Cd8b1* transcripts in cluster C12 separated by sample and treatment group. (C-E) RNA velocity vectors and Pdcd1 expression/velocity in UMAP space. (J) PAGA analysis; arrow thickness indicates transition likelihood between clusters. (F) Random walk analysis based on RNA velocity data using clusters C8 and C12 as origins, determined by CellRank 2. (G) Frequency of IFNγ^+ and IFNγ^+TNFα^+ of TCF1$^-$ or TCF1$^+$ P14 following *ex vivo* stimulation with gp33 peptide. Statistical significance was determined using a two-tailed paired t test. Ns $P > 0.05$.

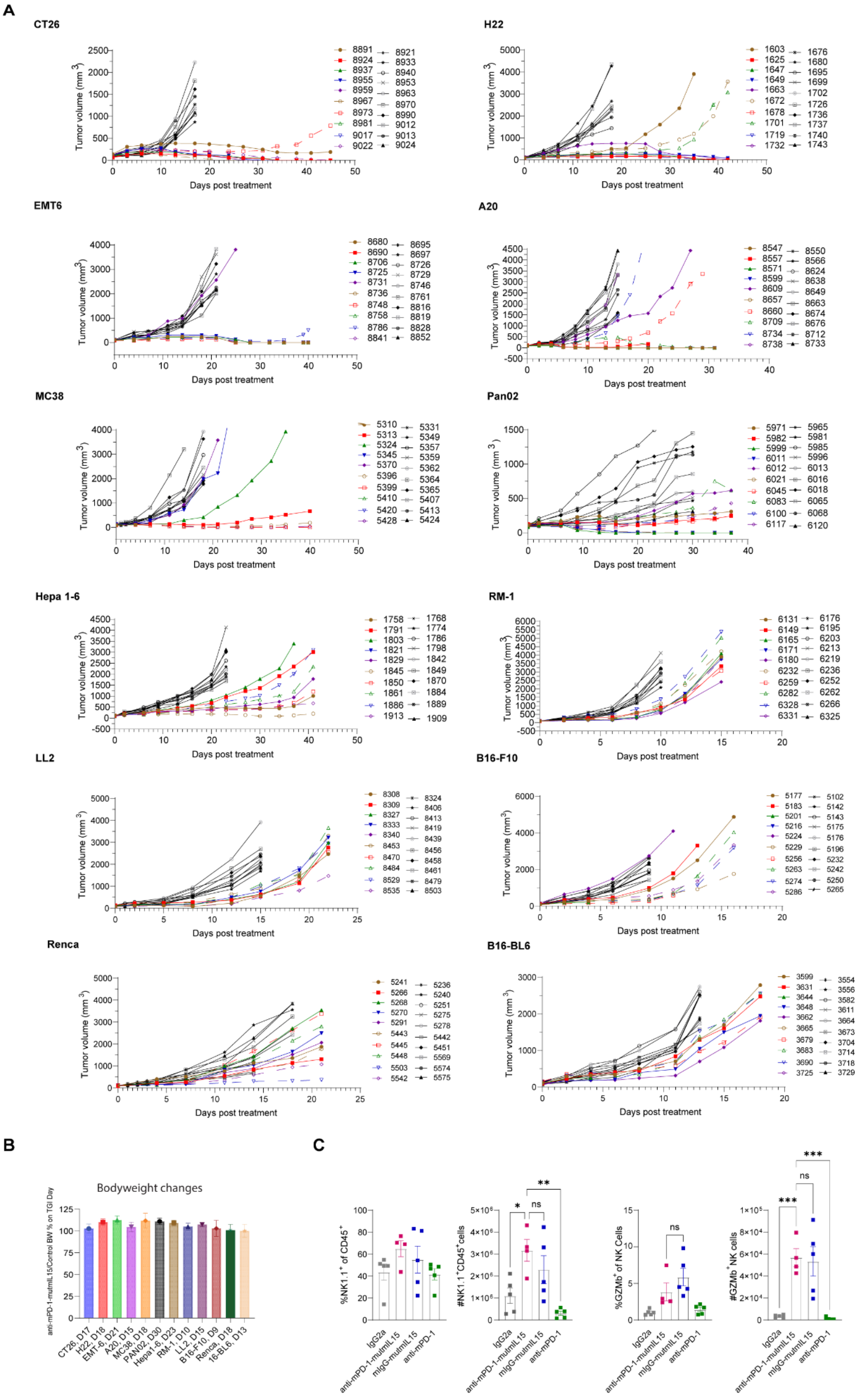
A
CT26
H22
EMT6
A20
MC38
Pan02
Hepa 1-6
RM-1
LL2
B16-F10
Renca
B16-BL6
Tumor volume (mm 3)
Days post treatment
B
Bodyweight changes
anti-mPD-1-mutmIL15/Control BW % on TGI Day
CT26, D17
H22, D18
EMT-6, D21
A20, D15
MC38, D18
PAN02, D30
Hepa1-6, D23
RM-1, D10
LL2, D15
B16-F10, D9
Renca, D18
16-BL6, D13
C
%NK1.1+ of CD45+
#NK1.1+CD45+cells
%GZMb+ of NK Cells
#GZMb+ NK cells
IgG2a
anti-mPD-1-mutmIL15
mIgG-mutmIL15
anti-mPD-1
ns
*
**
***

166

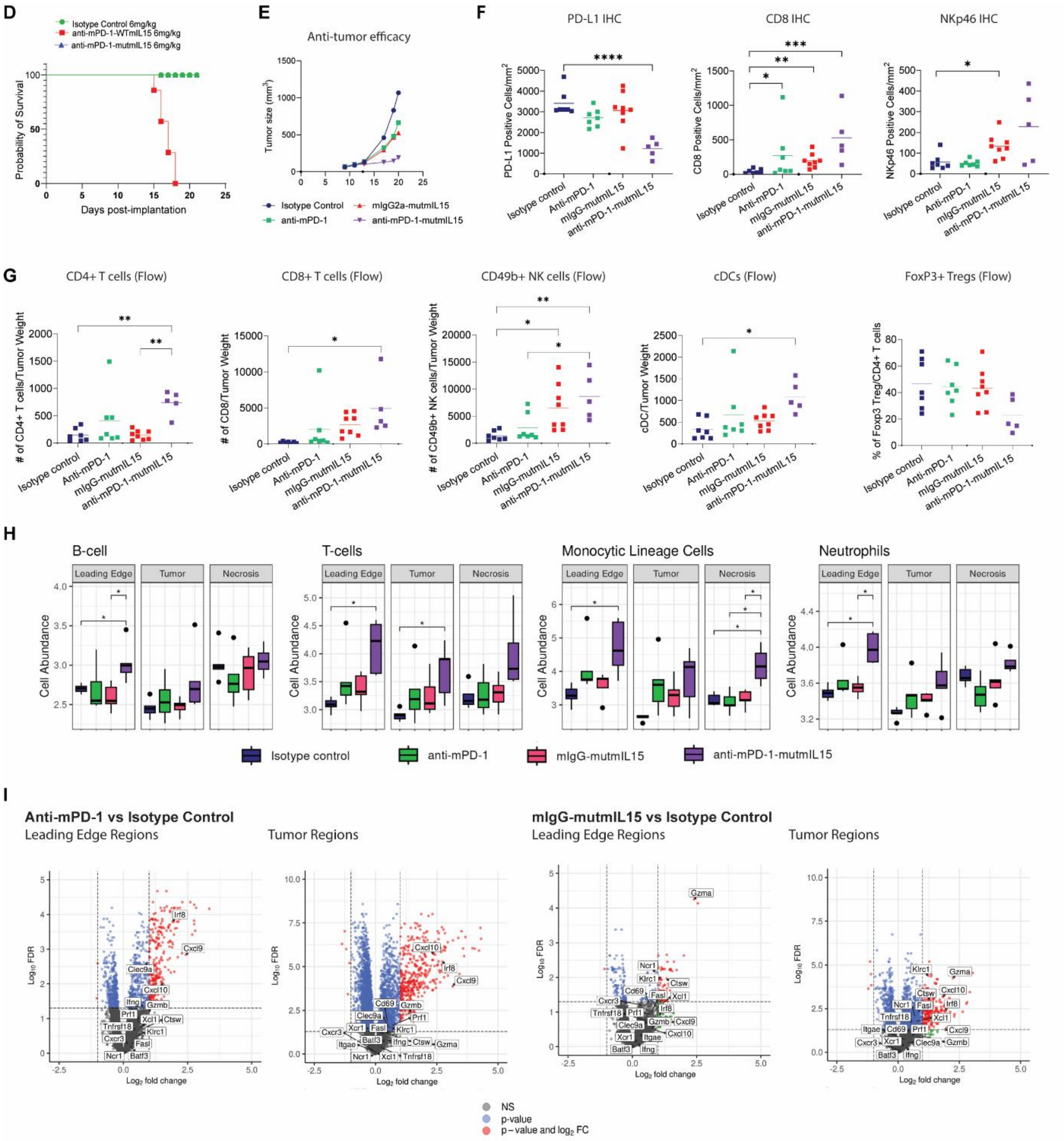
D
Isotype Control 6mg/kg
anti-mPD-1-WTmIL15 6mg/kg
anti-mPD-1-mutmIL15 6mg/kg
Probability of Survival
Days post-implantation
E
Anti-tumor efficacy
Tumor size (mm3)
Isotype Control
anti-mPD-1
mIgG2a-mutmIL15
anti-mPD-1-mutmIL15
F
PD-L1 IHC
CD8 IHC
NKp46 IHC
PD-L1 Positive Cells/mm2
CD8 Positive Cells/mm2
NKp46 Positive Cells/mm2
Isotype control
Anti-mPD-1
mIgG-mutmIL15
anti-mPD-1-mutmIL15
G
CD4+ T cells (Flow)
CD8+ T cells (Flow)
CD49b+ NK cells (Flow)
cDCs (Flow)
FoxP3+ Tregs (Flow)
# of CD4+ T cells/Tumor Weight
# of CD8/Tumor Weight
# of CD49b+ NK cells/Tumor Weight
cDC/Tumor Weight
% of Foxp3 Treg/CD4+ T cells
H
B-cell
T-cells
Monocytic Lineage Cells
Neutrophils
Leading Edge
Tumor
Necrosis
Cell Abundance
Isotype control
anti-mPD-1
mIgG-mutmIL15
anti-mPD-1-mutmIL15
I
Anti-mPD-1 vs Isotype Control
Leading Edge Regions
Tumor Regions
mIgG-mutmIL15 vs Isotype Control
Leading Edge Regions
Tumor Regions
Log10 FDR
Log2 fold change
NS
p-value
p – value and log2 FC

Fig. S5 Anti-tumor activity of SAR'877 surrogate in syngeneic mouse models and spatial transcriptomic characterization of the B16-F10-OVA biomarker study

(A) Tumor volume and (B) Percentage body weight change in mice from 12 different syngeneic tumor models (MuScreen) treated with anti-mPD-1-mutmIL15 vs. isotype control on tumor growth inhibition (TGI) day. (C) Frequency and total number of NK cells and Granzyme B expressing NK cells in MC38 tumor-bearing mice treated with anti-mPD-1-mutmIL15 vs. controls. Statistical significance was determined using one-way ANOVA followed by Dunnett's multiple comparisons test. * $P < 0.05$, ** $P < 0.01$, *** $P < 0.001$, **** $P < 0.0001$.

(D) Survival curves of MC38 tumor-bearing mice treated with anti-mPD-1-mutmIL-15, anti-mPD-1-WTmIL-15, or an isotype control. (E) Tumor size from the biomarker study conducted in the B16-F10-OVA tumor model. (F) Quantitative assessment of PD-L1, CD8 and NKp46 by immunohistochemistry in B16-F10-OVA treated tumors. Data are shown as scatter dot plots with mean. Statistical significance was assessed by one-way ANOVA followed by Dunnett's multiple comparisons test versus Isotype Control. * $P < 0.05$; ** $P < 0.01$; *** $P < 0.001$; *** $P < 0.001$. (G) Quantitative assessment of cell type abundances in dissociated B16-F10-OVA treated-tumors by flow cytometry. Data are shown as scatter dot plots with mean. Statistical significance was assessed by one-way ANOVA followed by Dunnett's multiple comparisons test versus anti-mPD-1-mutmIL15. * $P < 0.05$; ** $P < 0.01$. (H) GeoMx DSP cell type abundance (MCP-counter) per spatial compartment (Leading Edge, Tumor and Necrosis) in B16-F10-OVA treated-tumors. Data are shown as box plots with median and interquartile range. * $P < 0.05$ (I) GeoMx DSP volcano plot of differentially expressed genes (DEGs) in anti-mPD-1-treated or mIgG-mutmIL15-treated B16-F10-OVA tumors relative to Isotype Control in both leading edge and tumor regions.

Group comparisons were performed using Wilcoxon rank-sum tests with False Discovery Rate (FDR) correction. FDR < 0.05 was considered statistically significant.

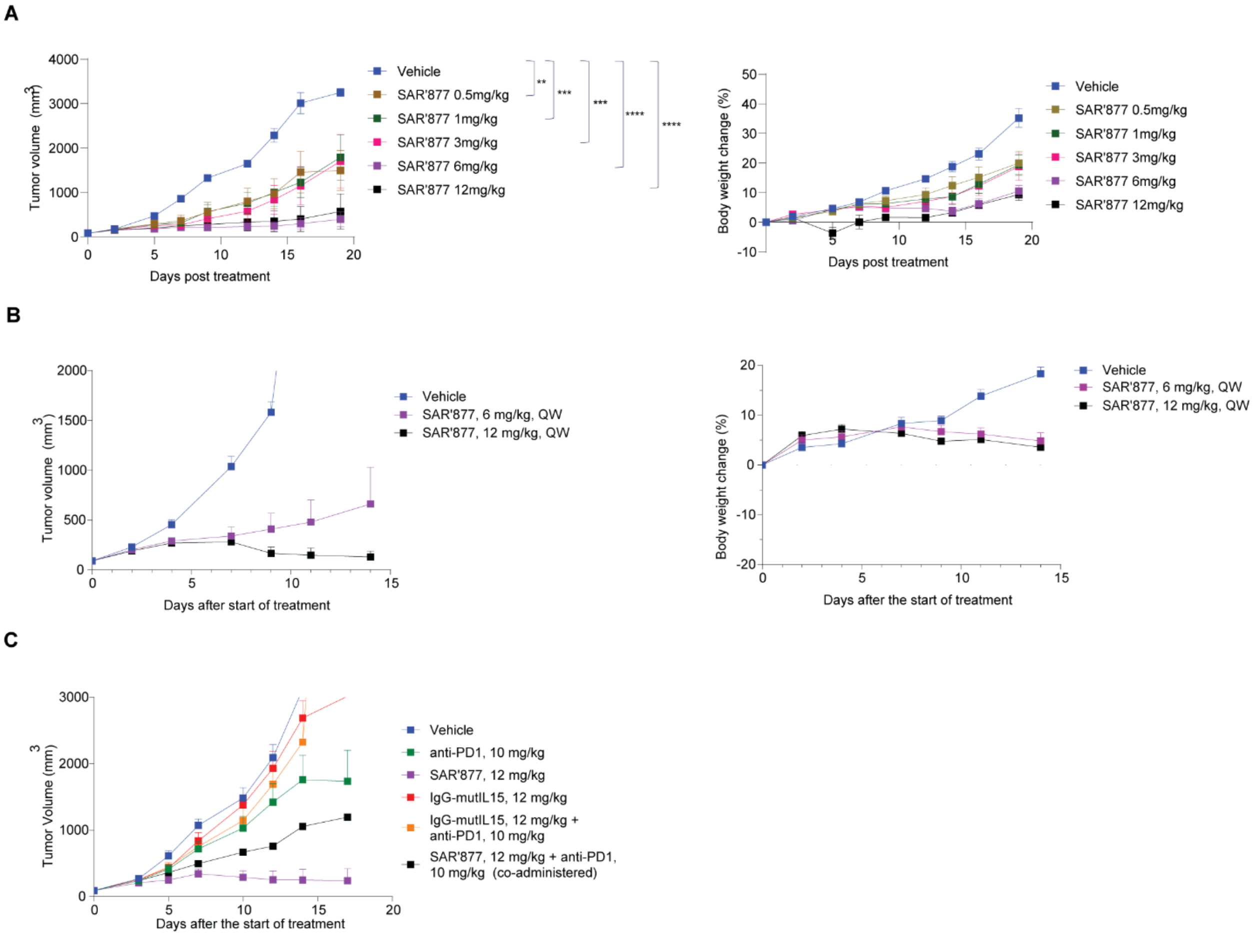

Fig. S6 Activity of SAR'877 in a human PD-1/PD-L1 transgenic mouse model

(A) SAR'877 was administered to huPD-(L)1-BALB/c mice bearing CT26-huPD-L1 tumors and tumor volume was monitored (single dose, IV, n=6 animals per group). Data is presented as mean± SD. Statistical analysis of difference in the tumor volume among the groups were conducted on the data obtained at Day 16 after treatment. A one-way ANOVA was performed to compare the tumor volume, and no significant F-statistics was obtained, thus comparisons between groups were carried out with Dunnett test (two sided). All data were analyzed using SPSS 17.0, $p < 0.05$ was considered to be statistically significant. Percentage body weight change is shown in the graph on

the right. (B) Mice bearing CT26-huPD-L1 tumors were treated with multiple doses of SAR'877 (QW dosing, IP, n=5 animals per group. Percentage body weight change is shown in the graph on the right. (C) Mice bearing CT26-huPD-L1 tumors were treated with multiple doses of SAR'877 (QW dosing, IP), anti-PD-1 (BIW dosing, IP), or IgG-mutIL15 (QW, IP), n=5-6 animals per group. Data are shown for each experiment until the date of termination of the vehicle control group.

Model	Cancer type	Media for cell culture	Cells inoculated	Mouse strain	Mouse sex
**EMT6**	Breast	DMEM+10%FBS	5×10^5	BALB/C	Female
**CT26.WT**	Colorectal	RPMI1640+10%FBS	5×10^5	BALB/C	Female
**MC38**	Colorectal	DMEM+10%FBS	1×10^6	C57BL/6J	Female
**Renca**	Kidney	DMEM+10%FBS	1×10^6	BALB/c	Female
**H22**	Liver	RPMI1640+10%FBS	1×10^6	BALB/C	Female
**Hepa 1-6**	Liver	DMEM+10%FBS	5×10^6	C57BL/6J	Female
**LL/2**	Lung	DMEM+10%FBS	3×10^5	C57BL/6J	Female
**A20**	Lymphoma	RPMI1640+10%FBS	5×10^5	BALB/C	Female
**B16-BL6**	Melanoma	RPMI1640+10%FBS	2×10^5	C57BL/6J	Female
**B16-F10**	Melanoma	DMEM+10%FBS	2×10^5	C57BL/6J	Female
**Pan02**	Pancreatic	RPMI1640+10%FBS	3×10^6	C57BL/6J	Female
**RM-1**	Prostate	RPMI1640+10%FBS	1×10^6	C57BL/6J	Male

Table S1. Anti-tumor efficacy in 12 syngeneic tumor models (MuScreen) details. Recapitulative table of 12 syngeneic tumor models used for the MuScreen, including model, cancer type, media for cell culture, number of cells inoculated, mouse strain and mouse sex information.

Internal

Group ID	No. of Animals	Treatment	Route	Dose (mg/kg)	Dosing Frequency
**1**	10	Isotype Control	IP	10	2QW (= 4 doses)
**2**	10	anti-mPD-1	IP	10	2QW (= 4 doses)
**3**	10	mIgG-mutmIL15	IV	1	QW (= 2 doses)
**4**	10	anti-mPD-1-mutmIL15	IV	1	QW (= 2 doses)

Table S2. Biomarker study in the B16-F10-OVA model, overview of treatment groups. IP: Intraperitoneal; IV: Intravenous; QW: Once Weekly; 2QW: Twice Weekly. Mice were pooled and randomly distributed to the control and treatment groups (10 mice per group), with tumor size ranging from 46 to 148 mm3. Anti-mPD-1-mutmIL15 was tested against isotype control, anti-mPD-1 and an untargeted murine IL-15 mutein (mIgG-mutmIL15).

Biological Functions	Leading Edge	Tumor
Cytotoxicity of lymphocytes	2.864	2.792
Cytotoxicity of natural killer cells	2.289	1.974
Cytotoxicity of T lymphocytes	2.054	2.2
Maturation of T lymphocytes	2.847	N/A
Maturation of natural killer T lymphocytes	2.392	N/A
T cell migration	5.192	3.492
NK cell migration	2.767	N/A
Proliferation of lymphocytes	3.037	1.317
NK cell proliferation	2.867	N/A
Proliferation of T lymphocytes	3.571	1.07
Proliferation of activated T lymphocytes	2.091	1.574
Proliferation of CD8+ T lymphocyte	2.798	N/A

Table S3. Comparison of Biological Functions, anti-mPD-1-mutmIL15 relative to anti-mPD-1. GeoMx DSP biological function enrichment analysis with spatial resolution identified by

Internal

Ingenuity Pathway Analysis (IPA) in B16-F10-OVA treated-tumors. The table shows the activation z-score (positive score = increase in function) of anti-mPD-1-mutmIL15 relative to anti-mPD-1 per spatial regions (Leading Edge and Tumor).
